# The Impact of Nutritional Status on Oral Health Outcomes and Management in Older Adults: A Systematic Review and Meta‐Analysis

**DOI:** 10.1111/ger.70030

**Published:** 2025-12-02

**Authors:** Kittipit Srisanoi, Ines Wnuk, Sabrina Maniewicz, Gerry McKenna, Frauke Müller, Claudio Leles, Piero Papi, Jayne Woodside, Murali Srinivasan

**Affiliations:** ^1^ Clinic of General, Special Care, and Geriatric Dentistry, Center for Dental Medicine University of Zurich Zurich Switzerland; ^2^ Faculty of Dentistry Khon Kaen University Khon Kaen Thailand; ^3^ Division of Gerodontology and Removable Prosthodontics, University Clinics of Dental Medicine University of Geneva Geneva Switzerland; ^4^ Center of Public Health Queen's University of Belfast Institute of Clinical Sciences Belfast UK; ^5^ Center of Excellence in Genomics and Precision Dentistry, International Program in Geriatric Dentistry and Special Care, Faculty of Dentistry Chulalongkorn University Bangkok Thailand; ^6^ Federal University of Goias Goiania Brazil; ^7^ Department of Oral and Maxillo‐Facial Sciences “Sapienza” University of Rome Rome Italy

**Keywords:** geriatric dentistry, malnutrition, meta‐analysis, nutrition, systematic review

## Abstract

**Objective:**

This systematic review and meta‐analysis evaluated the relationship between nutritional status and the oral health of older adults (aged 75 years or older). The PECO focus question was, ‘What is the occurrence, association and impact of nutritional state on the oral health outcomes and its management in older adults?’

**Methods:**

A comprehensive literature search was performed across MEDLINE (PubMed), Embase and the Cochrane Library. Publications up to April 2025 were considered, yielding 3324 records for initial screening. Quality assessment of the included studies predominantly revealed a low to moderate risk of bias.

**Results:**

Eighty‐three studies were included for data analysis. Meta‐analyses demonstrated that older adults at risk of malnutrition had fewer teeth (SMD = −0.29; 95% CI: −0.46, −0.11; *p* = 0.002). Those with < 20 teeth and who were not rehabilitated with dentures were more likely to be malnourished (OR = 4.00; 95% CI: 1.21, 13.18; *p* = 0.02). Malnutrition was associated with self‐reported chewing problems (OR = 2.38; 95% CI: 1.74, 3.26; *p* < 0.0001), swallowing problems (OR = 3.18; 95% CI: 2.20, 4.61; *p* < 0.0001) or dry mouth (OR = 2.35; 95% CI: 1.91, 2.88; *p* < 0.0001). Conversely, those with oral pain showed lower odds of malnutrition risk (OR = 0.68; 95% CI: 0.52, 0.89; *p* = 0.005).

**Conclusions:**

Fewer teeth, existing chewing or swallowing problems, dry mouth and the absence of removable dentures where needed, were associated with malnutrition in care‐dependent older adults. The current lack of longitudinal studies and proof of causality for malnutrition affecting oral health outcomes underscores the need for further research to better clarify the complex relationship between oral health and nutrition in older populations.

**Trial Registration:**

PROSPERO Registration: CRD420251003549.

## 1 Introduction

The global demographic change is driven by rising life expectancy, thanks to greater health awareness, improved healthcare systems and advances in medicine. However, ageing often comes with increasing frailty and dependency, both of which can significantly affect overall health and particularly nutritional status. The pooled prevalence of malnutrition among older adults ranges from 0.8% to 24.4%, depending on the region. It is more common in individuals living in homecare settings or rural areas [1]. As age and frailty progress, many older adults become more reliant on daily assistance, further impacting their nutritional well‐being. Evidence suggests that institutionalised and functionally dependent older adults tend to experience a decline in both oral health and nutrition [2], while community‐dwelling, independent individuals typically maintain better oral and nutritional status [3].

Oral health plays a crucial role in determining nutritional status, as it directly affects the ability to eat [4]. Dietary variety and overall intake can be limited by common oral problems in older adults, such as dental caries, periodontal disease and tooth loss [5]. A previous systematic review showed that posterior functional units, number of teeth and chewing deficiencies were linked to malnutrition [6, 7]. However, despite this association, several systematic reviews have explicitly shown unrelated results regarding whether prosthetic rehabilitation can actually improve nutritional outcomes [8, 9, 10]. This suggests that other oral health factors may also contribute to poor nutritional status in this population. In addition, general health decline, polypharmacy [11] and socioeconomic limitations [12] can all be associated. In turn, severe malnutrition may also influence oral health, for example, by a lower intake of nutrients essential for bone metabolism, saliva production, mucosal health or immune defence, but potential causality has been little studied [13, 14]. Altogether, these factors highlight the clinical importance of malnutrition, which is associated with increased mortality, infections and reduced quality of life [15].

With an increasingly ageing population, understanding this two‐way relationship between oral health and nutritional status is essential for guiding geriatric care. Exploring oral health factors beyond just tooth loss can give us a more complete picture of this complex interrelationship. This systematic review aims to critically assess current evidence on the relationship between nutritional status and oral health outcomes in older adults aged 75 years or older. Specifically, it examines the impacts of malnutrition and nutritional risk in relation to oral health problems—including a reduced number of teeth and denture wearing, chewing problems, swallowing problems, dry mouth and oral pain—and vice versa, to illustrate how different aspects of oral health impact nutritional status.

## 2 Materials and Methods

This systematic review and meta‐analysis was carried out and reported in accordance with the PRISMA (Preferred Reporting Items for Systematic Reviews and Meta‐Analyses) guidelines [16, 17]. The completed PRISMA checklist is provided as Data S1. The protocol of this review was registered with PROSPERO (the International Prospective Register of Systematic Reviews) under the registration number CRD420251003549.

### 2.1 Eligibility Criteria and Information Sources

The details of the inclusion and exclusion criteria for this systematic review, along with the sources of information used to explore relevant records, are described in Table 1. The final search was performed on April 17, 2025.

**TABLE 1:** Population information exposure outcome (PECO) table showing the focus question, inclusion criteria, information sources, search terms and the search strategy applied for this systematic review.

Focus question	What is the occurrence, association and impact of nutritional state on the oral health outcomes and its management in older adults?
PROSPERO	CRD420251003549
**Database search**	
Database search	MEDLINE (PubMed), Embase, the Cochrane Library
**Supplementary hand search**	
References	Included articles and identified reviews
**Grey literature search**	
Contact with experts	Authors of included articles, researchers with a known interest in gerodontology research
**Selection process**	
Inclusion criteria	Studies reporting on the effects of nutritional state and oral health outcomes in older adults aged 75 or older (mean age of participants ≥ 75 years) Sample size ≥ 10 participants per study group Subjects must have been clinically followed in recalls
Exclusion criteria	Topic not relevant to the focus question Ineligible study type (e.g., case reports, review articles, animal studies, in vitro study, study protocols, abstract only) Ineligible study design Irrelevant study outcomes Insufficient participant information, and no response from investigators when clarification was sought Previous investigations reporting on the same patient population (excluded, but retained for reference)
Identification process	Records were reviewed by at least two investigators independently, disagreements were resolved by discussion, and authors were contacted for clarification when required. Records in languages other than English that potentially fulfilled inclusion criteria were translated initially by the investigators, colleagues or ‘Google Translator’. No investigations met the inclusion criteria, and therefore no formal translations were completed.
Contact with authors	Research potentially met the inclusion criteria, but full text article was unavailable Research potentially met the inclusion criteria, but data reporting was incomplete or unclear Research identified through grey literature search
**Search terms**	
Population	#1 [MeSH]: ‘Aged’ OR **‘Dental Care for Aged’** #2 [Title/Abstract]: ‘Elder*’ OR ‘Geriatric Population*’ OR ‘Geriatric Patient*’ OR ‘Ageing Population’ OR ‘Aged Population’ OR ‘Aged 60’ OR ‘60 year*’ OR ‘Aged 75’ OR ‘75 year*’ OR ‘Aged 80’ OR ‘80 year*’ OR ‘Senior*’ OR ‘Care‐depend*’ OR ‘Older Adults’ OR ‘Older People’
Exposure	#3 [MeSH]: ‘Elder Nutritional Physiological Phenomena’ OR ‘Nutritional Assessment’ OR ‘Nutritional Status’ OR ‘Nutritional Surveys’ OR ‘Nutrition Disorders’ OR ‘Malnutrition’ OR ‘Body Mass Index’ OR ‘Obesity Paradox’ OR ‘Proteins’ #4 [Title/Abstract]: ‘Nutritional Risk Screening’ OR ‘Global Leadership Initiative on Malnutrition’ **OR** ‘Mini‐nutritional Assessment’ **OR** ‘Mini‐nutritional Assessment Short Form’ **OR** ‘Nutritional Biomarkers’ **OR** ‘Functional Index Measure’ **OR** ‘C‐reactive Protein Test’ **OR** ‘NRS’ OR ‘MNA’ OR ‘MNA‐SF’ OR ‘FIM’ OR ‘GLIM’ OR ‘BMI’ OR ‘CRP’
Comparison	No search term
Outcome	#5 [MeSH]: ‘Oral Health’ OR ‘Dental Health Surveys’ OR ‘Mouth Diseases’ **OR** ‘Jaw, Edentulous’ OR ‘Dental Caries’ **OR** ‘Periodontal Diseases’ OR ‘Dental pulp diseases’ #6 [Title/Abstract]: ‘**Tooth Loss’** OR ‘Edent*’ OR ‘**Missing Teeth’** OR ‘**Caries’** OR ‘Carious’ OR ‘Decay*’ OR ‘DMF‐T’ OR ‘Periodont*’ OR ‘Pulp Necrosis’ **OR** ‘Pulpitis’ **OR** ‘Nonvital’ **OR** ‘Non‐vital’ **OR** ‘Periapical’ **OR** ‘Pulpitis’ **OR** ‘Nonvital’ **OR** ‘Non‐vital’ **OR** ‘Periapical’ **OR** ‘**Oral Health‐Related Quality of Life’ OR** ‘**OHRQoL’ OR** ‘**Oral Health Impact Profile’ OR** ‘**OHIP’**
Filters	No language restriction
Search Builder	(#1 OR #2) AND (#3 OR #4) AND (#5 OR #6)
Search date	Completed on 17 April 2025

### 2.2 Search Strategy and Selection Process

The search terms were developed using the PECO (Population, Exposure, Comparison and Outcome) framework and combined with Boolean operators (OR, AND, NOT) to construct the search strategy. The first reviewer (K.S.) performed the initial search across all specified databases. Any issues identified during the process were corrected, and the search strategy was refined accordingly. The details of all search terms and the final strategy are provided in Table 1.

Search results from each database were imported into Rayyan (https://www.rayyan.ai), an online platform designed to facilitate systematic review management. The software automatically removed duplicate records as the initial step. Following this, two investigators (K.S. and I.W.) conducted screening of the title and abstract within the platform. To ensure consistency of the screening process, inter‐investigator reliability was evaluated using Cohen's unweighted kappa (κ) [18]. Following this step, the selected studies underwent full‐text review and data extraction, based on mutual agreement between the two investigators.

When full‐text articles were not available or lacked sufficient detail, especially for the mean age of participants, to ensure the correct inclusion and exclusion, the reviewers contacted with the corresponding author to provide more details. The reference cross‐checks and manual searches were performed to identify studies that were not discovered by the search strategy.

### 2.3 Data Collection Process and Data Items

Data on the association between nutrition and oral health outcomes were collected by consensus between two investigators (K.S. and I.W.), including study design, participant characteristics, number of participants, age, nutritional evaluation tools and outcome measures such as dentition status, number of teeth, prosthetic rehabilitation, dry mouth or hyposalivation, oral hygiene, oral frailty and oral problems. The intervention studies were extracted for additional information including details of the intervention and the follow‐up period. The main findings from each study were summarised focusing on the review's research question. Additionally, for studies that reported only the proportion of an event, the corresponding absolute number was calculated.

### 2.4 Study Risk of Bias Assessment

The risk of bias for the included association studies was simultaneously assessed by two investigators (K.S. and I.W.) using the Newcastle‐Ottawa Scale (NOS) and the NOS adapted for cross‐sectional studies [19, 20]. The included interventional studies were evaluated using the Cochrane tool for the assessment of the risk of bias (RoB 2) and Risk of Bias in Non‐randomised Studies of Interventions (ROBINS‐I) for randomised controlled trials (RCTs) and non‐RCTs, respectively [21].

### 2.5 Summary Measures and Synthesis of Results

A meta‐analysis was performed using Review Manager (RevMan) version 5.4.1 (The Nordic Cochrane Center, The Cochrane Collaboration, Copenhagen, Denmark). Odds ratio (ORs), along with their respective 95% confidence intervals (95% CIs), were used to explore the influence of the presence/absence of oral factors on the occurrence of malnutrition as characterised by the Mini Nutritional Assessment (MNA) or Mini Nutritional Assessment Short Form (MNA‐SF). The MNA is a widely validated tool in nutritional research. A score of ≥ 24 is classified as well‐nourished, 17–23.5 as at risk of malnutrition, and < 17 as malnourished. Similarly, the MNA‐SF has been validated against MNA with good sensitivity and specificity [22]. The MNA‐SF is a shortened version, scores of 12–14, 8–11 and ≤ 7 represent well‐nourished, at risk and malnourished, respectively, as in the MNA. Studies using other nutritional assessment tools were descriptively reported but not included in the meta‐analysis.

In addition, the Tau‐squared (Tau^2^), Chi‐squared (Chi^2^) and *I*‐squared (*I* ^2^) statistics were used to assess heterogeneity among studies. Random‐effects or a fixed‐effects model was applied to calculate the pooled effect estimates [23]. Heterogeneity among studies was further explored through subgroup analyses and sensitivity analyses were performed to assess the accuracy of the pooled results. Finally, the results were visually presented in a forest plot.

### 2.6 Publication Biases and Additional Analyses

A qualitative analysis was performed on all included studies to report their study design, participant characteristics, focus outcomes, nutritional evaluation tools and main conclusions. Additionally, the study of interventions was reported on intervention groups, specific intervention details, follow‐up duration, trial outcomes. Publication bias was planned to be evaluated across the studies for each analysis exploring graphically with funnel plots if more than 10 studies were included for the analysis [24].

## 3 Results

### 3.1 Study Selection

The search strategy detected 3688 records (MEDLINE [PubMed]: *n* = 913; Embase: *n* = 605; CENTRAL: *n* = 2170). The removal of duplicate records was automatically removed by the software, resulting in the elimination of 364 records. A total of 3324 records were screened for title and abstract screening. The list of 451 potentially included records was sought for retrieval, but 10 records were not retrieved. The final list of 441 records was further full‐text screened, of which 371 records were excluded due to ineligible participants age (*n* = 172), review articles (*n* = 58), ineligible study design (*n* = 55), irrelevant study outcomes (*n* = 27), abstracts only (*n* = 19), duplication (*n* = 8) and study protocols (*n* = 8). The authors of 26 records reporting an unclear mean age of participants were contacted by email for clarification, of which 2 records were included, 3 records were excluded due to ineligible participants age, and 21 records were excluded due to non‐response. The inter‐investigator reliability for the study screening process was almost perfect, with a kappa (*κ*) score 0.89 (95% CI: 0.882, 0.903).

**FIGURE 1:**
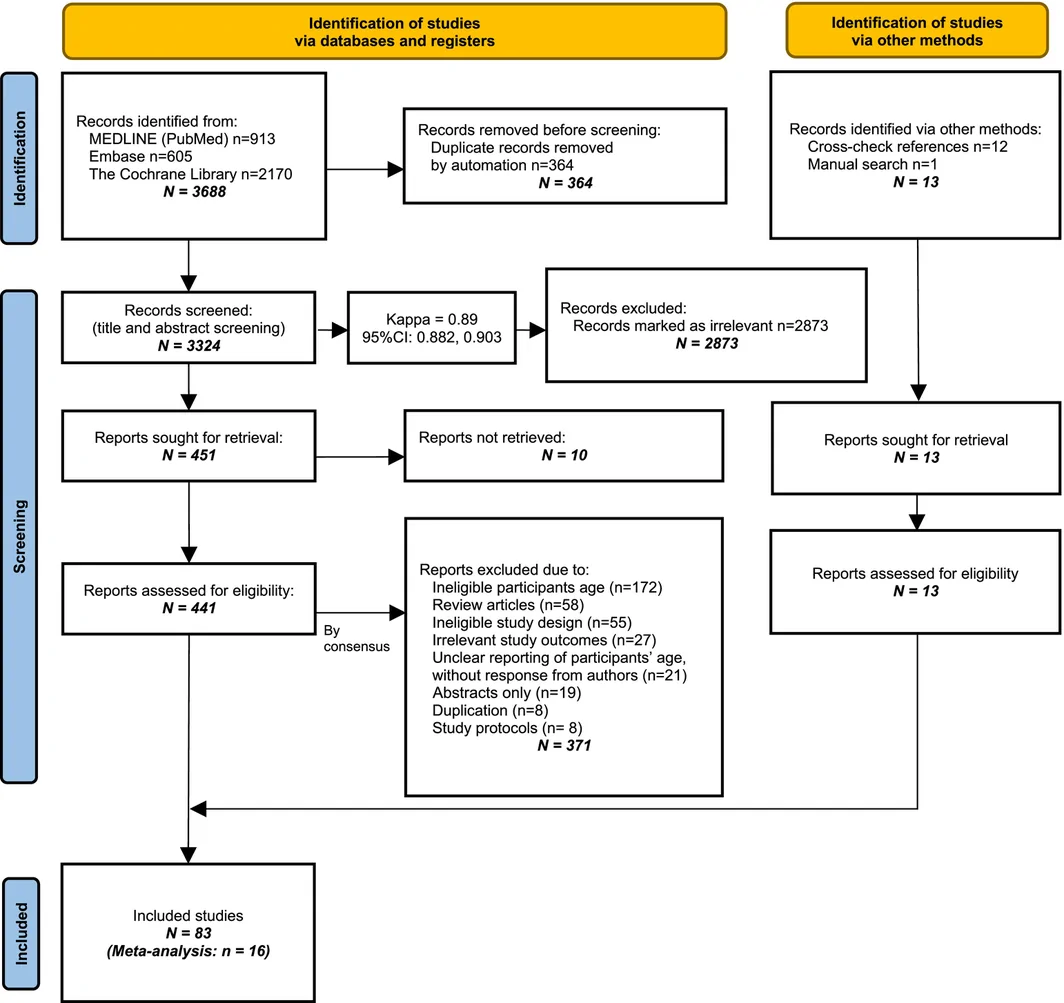
PRISMA flow diagram summarising the study selection process, including records identified, duplicates removed, records screened and studies included in the meta‐analysis. *N* or *n*, number. [Colour figure can be viewed at wileyonlinelibrary.com]

Finally, 70 studies related to the association between nutrition and oral health outcomes in older adults were included. Additionally, 12 records were identified by a cross‐check of the references and 1 record was identified by manual search. A final list of 83 records was qualified for data extraction and final analysis. Of the 83 included studies, 57 association studies [25, 26, 27, 28, 29, 30, 31, 32, 33, 34, 35, 36, 37, 38, 39, 40, 41, 42, 43, 44, 45, 46, 47, 48, 49, 50, 51, 52, 53, 54, 55, 56, 57, 58, 59, 60, 61, 62, 63, 64, 65, 66, 67, 68, 69, 70, 71, 72, 73, 74, 75, 76, 77, 78, 79, 80, 81, 82] and 7 RCTs [83, 84, 85, 86, 87, 88, 89] were descriptively analysed, 16 studies were deemed eligible for a meta‐analysis [90, 91, 92, 93, 94, 95, 96, 97, 98, 99, 100, 101, 102, 103, 104, 105], based on their study design and the information provided. The study selection process is demonstrated in a PRISMA flow diagram (Figure 1). Details of the excluded studies with the reasons for their exclusion, are clarified in Data S2.

### 3.2 Study Characteristics

The majority of included studies were conducted among care‐dependent older adults living in nursing homes, receiving long‐term care services or requiring special care such as palliative or home care, while the number of studies recruiting community‐dwelling participants was slightly lower. A smaller number of studies were conducted among hospitalised older adults in geriatric outpatient clinics or rehabilitation wards. Two studies included participants from multiple types of residential settings. Many studies used a cross‐sectional design; only six studies were prospective cohorts.

Most studies used the MNA or MNA‐SF as a screening tool to evaluate nutritional status. A smaller number used other screening methods, such as the Malnutrition Universal Screening Tool (MUST), Nutritional Screening Tool (NST), Nutritional Risk Screening (NRS), Geriatric Nutritional Risk Index (GNRI) or locally developed tools. In addition, some studies assessed nutritional status using anthropometric indicators (e.g., mid‐upper arm circumference), nutrient intake or nutritional biomarkers. Only two studies used a diagnostic tool, namely the Global Leadership Initiative on Malnutrition (GLIM) criteria. Associations between oral health and nutrition were reported using group comparisons, regression models and correlation analyses.

### 3.3 Synthesis of Results: Descriptive Analysis of the Included Studies

#### 3.3.1 Number of Teeth and Removable Dental Prosthesis

Studies examining the number of remaining natural teeth showed consistent results; 12 studies reported that the number of teeth was not significantly associated with inadequate nutrition. Functional occlusal units also showed a non‐significant association with poor nutrition in 7 studies, except for studies by Gil‐Montoya et al. [94] and El Osta et al. [35] It is interesting to note that Wakabayashi et al. [78] proposed an indirect link between malnutrition and occlusal support through issues with swallowing. However, a long‐term study by Kotronia et al. [49] discovered that a persistently poor diet was linked to fewer teeth and more oral health issues. For edentate older patients regardless of denture use, 4 studies found no significant association with nutritional status, but two studies by Krzyminska‐Siemaszko et al. [50] and Boulos et al. [92] found a significant association. Apparently, 5 studies reported that edentulous patients who were not wearing dentures were associated with poor nutritional status [27, 41, 65, 67, 106].

Five studies on patients wearing removable dentures, including all types, reported no significant association with nutritional status; only the studies by Kikutani et al. [107] reported a significant association with the risk of malnutrition, and Ritchie et al. [63] found a significant association with BMI and albumin levels. However, 3 studies found significant associations when denture‐needed participants were not using any type of denture [27, 91, 100]. Moreover, Mann et al. [74] found that not wearing dentures was significantly associated with compromised dietary intake, Similarly, longitudinal studies by Sadamori et al. [68] in older adults with dementia after 2 years of follow‐up showed comparable findings.

#### 3.3.2 Chewing Problems

Chewing problems were more consistently associated with nutritional status than the number of teeth. Nine studies reported a significant association with poor nutrition, but 4 studies by Bakker et al. [28], Van Der Pols‐Vijlbrief et al. [77], Liang et al. [51] and Davino et al. [32] reported no association. Furthermore, longitudinal studies with 2 years of follow‐up by Won et al. [103] and Holst et al. [42] found a significant association with undernutrition. Specifically, eating problems reported by 3 studies regarding tooth loss affecting food intake and oral dryness when eating showed a significant association with nutritional status [33, 43, 69]. Moreover, Nishio et al. [57] showed a significant association between uncomfortable eating in front of others and malnutrition diagnosed by GLIM criteria.

#### 3.3.3 Swallowing Problems

Swallowing problems were measured by different methods. Eight studies on subjective swallowing problems measured by EAT‐10 or simple questionnaires showed consistent results with a significant association with poor nutrition, except for the study by Van Der Pols‐Vijlbrief et al. [77] Moreover, the study by Nishio et al. [57] showed a significant association with malnutrition diagnosed by GLIM criteria. Objective swallowing problems measured by the modified water swallow test showed a non‐significant association in 3 studies, but the study by Kikutani et al. [100], using the cervical auscultation test, showed a significant association. Poisson et al. [61] and Ambrosio‐Palma et al. [90] found that reduced salivary flow and xerostomia, respectively, were associated with dysphagia, which was linked to poor dietary intake and nutritional status.

#### 3.3.4 Dry Mouth

Dry mouth showed conflicting associations with nutritional status. Four studies reported a significant association between self‐reported dry mouth and poor nutrition, and a study by Syrjälä et al. [26] emphasised the association with salivary flow rate, whereas 3 studies reported no significant association. El Osta et al. [35] measured clinical dry mouth in dementia patients and also showed no significant association with nutritional status. Poisson et al. [61] emphasised the non‐significant association with salivary hypofunction.

#### 3.3.5 Oral Frailty and Hypofunction

Regarding oral frailty, two studies by Iwasaki et al. [44] and Nakagawa et al. [64] found significant associations with poor nutrition. A longitudinal study by Iwasaki et al. [45] emphasised the decline in nutritional status in patients with oral frailty after 2 years of follow‐up. Ohta et al. [81] found a significant correlation between oral hypofunction and nutritional risk screened by MNA‐SF and NRS, but not with malnutrition diagnosed by GLIM criteria. Three studies reported a significant association with tongue pressure, but 3 studies showed no significant association. Gao et al. [97] reported a significant association with occlusal force, as well as Nishio et al. [57] using GLIM criteria.

#### 3.3.6 Oral Health and Hygiene

Four studies using the Geriatric Oral Health Assessment Index (GOHAI) found no significant association with poor nutrition, except for the study by Wu et al. [79] Inconclusively, each of two studies using similar measurements, including the Oral Health Assessment Tool (OHAT), the Revised Oral Assessment Guide (ROAG) and the Oral Health Impact Profile (OHIP‐14), found conflicting associations. In addition, Bakker et al. [28] and Koury et al. [99] reported no significant association between oral pain and malnutrition.

Oral hygiene measured by the tongue coating index (TCI) in the study by Fukuyama et al. [80] was significantly associated with nutritional status, but Izumi et al. [47] found no association. Moreover, two studies measured the plaque index (PI) and showed no significant association. Candidiasis was found to be associated with poor nutritional status in studies by Holst et al. [42] and Paillaud et al. [34], but not in the study by Poisson et al. [61].

All included studies are reported descriptively in Table 2.

**TABLE 2:** Summary of included studies for descriptive analysis.

Author/Year	Study design	Population	Age (years)	Study groups	Number of participants (*n*)	Focus outcomes	Nutritional evaluation tools	Main findings
**1. Studies grouped by nutritional status**								
Bakker 2018	Cross‐sectional	Community‐dwelling older adults	80.0 ± 1.0	**Group 1:** malnutrition **Group 2:** well‐nourished	**Group 1:** 49 patients **Group 2:** 973 patients	Edentulism, remaining teeth; implant‐supported overdenture; health‐related quality of life	The Guidelines of the Dutch Steering Group	Poorer health‐related quality of life was significantly associated with malnutrition. However, edentulism and oral health problems were not significantly associated with malnutrition.
Chiesi 2019	Cross‐sectional	Institutionalised older adults	84.4 ± 8.3	**Group 1:** low malnutrition risk **Group 2:** moderate malnutrition risk **Group 3:** high malnutrition risk	**Group 1:** 133 patients **Group 2:** 19 patients **Group 3:** 23 patients	GOHAI; ROAG; OHAT	MUST	Oral health and good nutrition appeared to have lower importance for quality of life compared to dignity, self‐esteem and social inclusion. However, oral health was not significantly associated with nutritional status.
Dewake 2017	Cross‐sectional	Older adults receiving day care service	80.4 ± 6.5	**Group 1:** malnutrition/at risk **Group 2:** well‐nourished	**Group 1:** 27 patients **Group 2:** 26 patients	Number of teeth; denture use	MNA‐SF	The number of teeth was significantly different between two groups, but denture use status was no significant.
El Helou 2014	Cross‐sectional	Hospitalised older adults	76.2 ± 5.6	**Group 1:** well‐nourished **Group 2:** malnutrition/at risk	**Group 1:** 65 patients **Group 2:** 50 patients	GOHAI; number of teeth; tooth loss; denture use; dry mouth	MNA	A high number of GOHAI score and not wearing a dental prosthesis were significantly associated with nutritional deficit, but there was no association with edentulism or xerostomia.
El Osta 2022	Cross‐sectional	Institutionalised older adults with dementia	83.9 ± 8.74	**Group 1:** inadequate nutrition **Group 2:** adequate nutrition	**Group 1:** 25 patients **Group 2:** 78 patients	PFUs; dry mouth	MUAC	Older adults with dementia who had ≤ 4 PFUs exhibited signs of malnutrition 7.5 times more compared to those with > 4PFUs, whereas dry mouth was not significantly associated. The number of PFUs was not significantly considered in determining the texture of food provided, suggesting a disconnect between oral health status and dietary accommodations.
Feldblum 2007	Cross‐sectional	Institutionalised and home‐dwelling older adults	75.0 ± 5.8	**Group 1:** malnutrition **Group 2:** at risk of malnutrition	**Group 1:** 48 patients **Group 2:** 211 patients	Chewing problems; swallowing problems; artificial teeth	MNA	Chewing problems were significantly more prevalence and associated with malnourished group, while at‐risk group had significantly more artificial teeth.
Forcano‐Sanjuan 2018	Cross‐sectional	Community‐dwelling older adults at high risk of hospital admission	79.9 ± 10.9	**Group 1:** at risk of malnutrition **Group 2:** well‐nourished	**Group 1:** 355 patients **Group 2:** 436 patients	Denture use; dysphagia	MNA‐SF	Patients at risk of malnutrition had a significantly higher prevalence of dysphagia, but there was no significant different in denture use.
Fukuyama 2024	Cross‐sectional	Older adults requiring long‐term care and receiving home‐visit dental care	85 (76–91)	**Group 1:** well‐nourished **Group 2:** malnutrition/at risk	**Group 1:** 25 patients **Group 2:** 85 patients	Number of teeth; occlusal support; Eichner Index; number of functional teeth; TCI; rinsing ability	MNA‐SF; SMI	The number of teeth, tongue pressure and PFUs were not significantly associated with nutritional status. Higher TCI was significantly associated with lower MNA‐SF scores.
Holst 2013	Prospective Cohort (1‐year follow‐up)	Hospitalised older adults	81.0 ± 7.6	**Group 1:** not at risk of malnutrition **Group 2:** at risk of malnutrition	233 patients	Chewing difficulties; taste disturbance; fungus in mouth	MUST; MNA; NRS‐2002	Nutritional risk among hospitalised older adults ranged from 47% to 68%, depending on the screening tool and setting. NRS‐2002 is recommended for initial screening. Chewing difficulties and oral fungus were associated with nutritional risk.
Hua 2022	Cross‐sectional	Institutionalised older adults	80.6 ± 9.0	**Group 1:** well‐nourished **Group 2:** at risk of malnutrition	**Group 1:** 261 patients **Group 2:** 125 patients	Tooth loss affecting food intake; BMI	MNA‐SF	Factors such as advanced age, presence of sarcopenia, tooth loss impacting food intake, reduced self‐care ability and limited dietary diversity were associated with higher nutritional risk.
Izumi 2020	Cross‐sectional	Institutionalised older adults	82.2 ± 9.8	**Group 1:** well‐nourished **Group 2:** malnutrition/at risk	**Group 1:** 14 patients **Group 2:** 38 patients	Swallowing problems; FTUs: number of teeth: tongue pressure; tongue stain; plaque index	MNA‐SF	Oral and pulmonary functions, as indicated by decreased tongue pressure and peak expiratory flow rate, were linked to poorer nutritional status in older adults residing in nursing homes.
Kucuk and Kapucu 2017	Cross‐sectional	Institutionalised older adults	78.7 ± 7.9	**Group 1:** malnutrition **Group 2:** at risk of malnutrition **Group 3:** well‐nourished	**Group 1:** 83 patients **Group 2:** 137 patients **Group 3:** 88 patients	Oral health problems (e.g., lacked teeth, dentures problems, dry mouth, mucositis); swallowing problems	MNA	The presence of oral health problems was significantly different, and swallowing problems were significantly associated with MNA score.
Lindmark 2018	Retrospective	Older adults with a care contact (municipal care or country council care)	82.8 ± 7.9	**Group 1:** well‐nourished **Group 2:** at risk of malnutrition **Group 3:** malnutrition	**Group 1:** 443 patients **Group 2:** 516 patients **Group 3:** 197 patients	ROAG‐J; presence of dentures or implants; fewer than 12 teeth	MNA	Issues related to voice and swallowing according to ROAG‐J items, were significantly associated with an increased risk of malnutrition.
Lopez‐Jornet 2013	Cross‐sectional	Institutionalised and non‐institutionalised older adults	75.7 ± 7.8	**Group 1:** malnutrition **Group 2:** at risk of malnutrition **Group 3:** well‐nourished	**Group 1:** 20 patients **Group 2:** 78 patients **Group 3:** 367 patients	Complete edentulism; number of teeth; number of missing teeth; denture use	MNA	Higher age and institutionalisation were significantly associated with a greater risk of malnutrition. There was no significant association between denture use or edentulism and nutritional risk.
Nishio 2024	Cross‐sectional	Community‐dwelling older adults aged ≥ 85 years receiving long‐term care	87.4 ± 2.3	**Group 1:** normal nutritional status **Group 2:** malnutrition	**Group 1:** 430 patients **Group 2:** 89 patients	Number of teeth; GOHAI; maximum occlusal force; saliva (mL/3 min); denture use	GLIM	Significant associations with malnutrition were observed for decline of maximum occlusal force, enjoyment of meals, swallowing problems, aesthetic problems and uncomfortable eating in front of others.
Nykänen 2013	Cross‐sectional	Community‐dwelling older adults aged ≥ 75 years	81.0 ± 4.6	**Group 1:** malnutrition/at risk **Group 2:** well‐nourished	**Group 1:** 106 patients **Group 2:** 590 patients	Dry mouth and chewing problems	MNA‐SF	Those with dry mouth or chewing problems were more likely to be malnourished or at risk of malnutrition.
Soini 2005	Cross‐sectional	Community‐dwelling older adults receiving public home care services	83.7 ± 4.4	**Group 1:** malnutrition/at risk **Group 2:** well‐nourished	**Group 1:** 24 patients **Group 2:** 27 patients	Oral health status (dry mouth, dentition status, chewing/swallowing difficulties) and related eating problems	MNA; biochemical and anthropometric indicators	Those with dry mouth and chewing/swallowing difficulties were significantly associated with lower MNA scores. Patients with functional natural dentition had a higher BMI than others.
Stratidaki 2024	Cross‐sectional	Community‐dwelling older adults receiving home care services	76.8 ± 6.7	**Group 1:** well‐nourished **Group 2:** at risk of malnutrition **Group 3:** malnutrition	**Group 1:** 379 patients **Group 2:** 205 patients **Group 3:** 146 patients	OHAT	MNA‐SF	Poor nutritional status in older adults was not significantly associated with poor oral health.
van der Pols‐Vijlbrief 2014	Cross‐sectional	Community‐dwelling older adults	81.7 ± 7.6	**Group 1:** well‐nourished **Group 2:** at risk of malnutrition **Group 3:** malnutrition	**Group 1:** 184 patients **Group 2:** 24 patients **Group 3:** 92 patients	Oral health; oral problems (e.g., swallowing and chewing problems, dry mouth); chewing surface; problems in eating; denture use	SNAQ	Only dry mouth was significantly associated with malnutrition.
Zenthöfer 2015	Cross‐sectional	Institutionalised older adults	83.2 ± 9.1	**Group 1:** well‐nourished **Group 2:** malnutrition/at risk	**Group 1:** 222 patients **Group 2:** 33 patients	Edentulism; denture status	BMI	Older adults with edentulism without wearing dentures and inadequate dental substitution were associated with higher risk of malnutrition.
**2. Studies grouped by oral health outcomes**								
**2.1 Number of teeth and removable dental prosthesis**								
Han and Kim 2016	Cross‐sectional	Edentulous older adults in one or both jaws	75.1 ± 6.1	**Group 1:** denture wearers **Group 2:** non‐denture wearers	**Group 1:** 1026 patients **Group 2:** 142 patients	Nutritional status and quality	Nutrient intake; EER	Non‐complete denture wearers were associated with the risk of undernourishment.
Julkunen 2024	Cross‐sectional	Institutionalised older adults	**Group 1:** 84.0 ± 7.0 **Group 2:** 83.0 ± 9.0	**Group 1:** edentulous **Group 2:** dentate with oral disease burden	**Group 1:** 94 patients **Group 2:** 209 patients	Nutritional status	MNA	Dentate individuals with high oral disease burden had similar odds of experiencing oral frailty as edentate residents. Both groups were not significantly different in nutritional status.
Krzyminska‐Siemaszko 2016	Cross‐sectional	Community‐dwelling older adults	77.4 ± 8.0	**Group 1.1:** edentulous **Group 1.2:** non‐edentulous **Group 2.1:** denture wearers **Group 2.2:** non‐denture wearers	**Group 1.1:** 1734 patients **Group 1.2:** 1894 patients **Group 2.1:** 2704 patients **Group 2.2:** 185 patients	Nutritional status	MNA‐SF	Factors independently associated with poor nutritional status included being female, advanced age, presence of depressive symptoms, cognitive impairment, multiple chronic diseases, anaemia and complete tooth loss.
Lamy 1999	Cross‐sectional	Institutionalised older adults	81.0 ± 8.0	**Group 1:** edentulous without dentures or with single complete denture **Group 2:** edentulous with two complete dentures **Group 3:** dentate with or without partial dentures	**Group 1:** 27 patients **Group 2:** 46 patients **Group 3:** 47 patients	Nutritional status	MNA; Questionnaire on eating habits (eating pleasure, difficulty eating hard foods and mashed food consumption)	Older individuals without dentures or with single complete denture had significantly lower MNA. In contrast, edentulous subjects with two complete dentures had better nutritional outcomes, reported fewer difficulties and enjoyed eating more. Poor masticatory capacity had also significant impact on nutritional status and increased mashed food intake.
Papas 1998	Cross‐sectional	Community‐dwelling older adults	75.2	**Group 1:** two complete dentures **Group 2:** single complete denture **Group 3:** partial dentures **Group 4:** dentate	181 patients	Nutritional quality	Nutrient intake	Degree of edentulousness correlated with quality of nutrient intake. The dentate group had significantly higher nutritional quality than the others.
Saarela 2014	Cross‐sectional	Institutionalised older adults	**Group 1:** 82.0 ± 7.8 **Group 2:** 82.0 ± 7.8 **Group 3:** 81.6 ± 7.8	**Group 1:** edentulous without denture **Group 2:** edentulous with some removable denture in one or both jaws **Group 3:** all or some natural teeth left with or without removable denture in one or both jaws	**Group 1:** 94 patients **Group 2:** 614 patients **Group 3:** 661 patients	Nutritional status	MNA	Edentulous individuals without dentures were associated with malnutrition, experienced oral symptoms like chewing and swallowing difficulties, and less frequently used oral care services. They were also more likely to consume puréed or soft food.
Saarela 2016	Cross‐sectional	Institutionalised older adults	83.0 ± 7.3		**Group 1:** 28 patients **Group 2:** 134 patients **Group 3:** 181 patients	Nutritional status	MNA	Edentulous individuals without dentures had significantly poorer nutritional status and lower protein intake, as well as a higher prevalence of chewing and swallowing difficulties.
Sadamori 2012	Prospective cohort (2‐year follow‐up)	Older women living in a nursing home, with and without dementia	86.6 ± 6.3–88.7 ± 7.6	**Group 1:** dementia with complete dentures **Group 2:** dementia without complete dentures **Group 3:** dementia with complete denture in 2006, without in 2008 **Group 4:** without dementia with complete denture **Group 5:** without dementia with complete denture in 2006, without in 2008	63 patients	Nutritional status	BMI; serum albumin level	Over a two‐year period, older women with dementia who did not wear dentures showed a significant decline in their daily calorie intake, while BMI and serum albumin levels remained stable. In contrast, those with dentures maintained a more consistent caloric intake.
**2.2 Chewing problems**								
Mann 2013	Cross‐sectional	Community‐dwelling older adults	75.5 ± 8.4	**Group 1:** chewing difficulties **Group 2:** swallowing difficulties **Group 3:** chewing and swallowing difficulties	**Group 1:** 113 patients **Group 2:** 51 patients **Group 3:** 11 patients	Nutritional quality	Nutrient intake	The three groups showed no significant differences in daily nutrient intake.
Wakabayashi 2018	Cross‐sectional	Institutionalised, hospitalised and community‐dwelling older adults	83 ± 8	**Group 1:** non‐functional occlusal support **Group 2:** functional occlusal support	**Group 1:** 138 patients **Group 2:** 216 patients	Nutritional status	MNA‐SF	Nutritional status was significantly different between both groups. Occlusal support was directly associated with dysphagia and indirectly associated with malnutrition via swallowing problems.
**2.3 Swallowing problems**								
Poisson 2016	Cross‐sectional	Hospitalised older adults in an acute geriatric care unit	85.3 ± 5.7	**Group 1:** with dysphagia **Group 2:** without dysphagia	**Group 1:** 34 patients **Group 2:** 122 patients	Nutritional status	MNA‐SF	Dysphagia, candidiasis, reduced salivary flow and PFUs were not significantly associated with malnutrition, only autonomy for oral care showed a significant association.
**2.4 Dry mouth**								
Iwasaki 2016	Cross‐sectional	Community‐dwelling older adults aged 80 years	80	**Group 1:** SSFR ≥ 0.5 mL/min **Group 2:** SSFR < 0.5 mL/min	**Group 1:** 316 patients **Group 2:** 36 patients	Nutritional status and quality	Anthropometric indicators; nutrient intake	Hyposalivation was associated with poorer dietary intake among older adults. This condition may negatively impact geriatric nutrition.
**2.5 Oral frailty and hypofunction**								
Iwasaki 2020	Cross‐sectional	Community‐dwelling older adults	77	**Group 1:** without oral frailty **Group 2:** with oral frailty	**Group 1:** 839 patients **Group 2:** 215 patients	Nutritional status	MNA‐SF; serum albumin level; BMI	Oral frailty was significantly associated with nutritional status indicated by MNA‐SF and serum albumin level.
Iwasaki 2021	Prospective cohort (2‐year follow‐up)	Community‐dwelling older adults	76.4	**Group 1:** without oral frailty **Group 2:** with oral frailty	**Group 1:** 399 patients **Group 2:** 67 patients	Nutritional status	MNA‐SF; BMI	Older adults with oral frailty experienced an increased risk of a decline in their nutritional status over time.
Nakagawa 2024	Cross‐sectional	Community‐dwelling older adult	79.9 ± 4.3	**Group 1:** with oral frailty **Group 2:** without oral frailty	**Group 1:** 1208 patients **Group 2:** 1519 patients	Nutritional status and quality	SNAQ; DVS; BMI	Oral frailty was associated with decreased appetite and dietary variety in late‐stage older adults.
**2.6 Oral health and hygiene**								
Chew 2023	Cross‐sectional	Hospitalised older adults	79.2 ± 8.3	**Group 1:** low risk ROAG **Group 2:** moderate risk ROAG **Group 3:** high risk ROAG	**Group 1:** 343 patients **Group 2:** 100 patients **Group 3:** 22 patients	Malnutrition risk	NST	Poor oral health was significantly associated with malnutrition, frailty and functional decline.
Galesi‐Pacheco 2021	Cross‐sectional	Institutionalised older adults	77.0 ± 10.0	**Group 1:** without oral problem **Group 2:** with oral problem	**Group 1:** 54 patients **Group 2:** 96 patients	Nutritional status	Nutrient intake; anthropometric indicators (e.g., BMI, MUAC)	Oral health issues lead to less consumption of energy, protein and lipids compared to those without such problems. Lower anthropometric measurements were exhibited, indicating a potential link between oral health and nutritional status.
Saarela 2019	Cross‐sectional	Institutionalised older adults	83.9	**Group 1:** without oral symptom **Group 2:** one oral symptom **Group 3:** two or three oral symptoms	**Group 1:** 1392 patients **Group 2:** 609 patients **Group 3:** 400 patients	Nutritional status	MNA	A higher oral symptom burden (chewing and swallowing difficulties and dry mouth) was associated with poorer nutritional status, reduced food intake, lower BMI and lower health‐related quality of life.
Shiraishi 2017	Retrospective	Older adults admitted to convalescent rehabilitation wards	80.5 ± 6.8	**Group 1:** without oral problem **Group 2:** with oral problem	**Group 1:** 16 patients **Group 2:** 92 patients	Nutritional status	MNA‐SF	ROAG score was negatively correlated with MNA‐SF score.
Paillaud 2004	Cross‐sectional	Hospitalised older adults in a geriatric rehabilitation unit	82.1 ± 8.6	**Group 1:** without oral candidiasis **Group 2:** with oral candidiasis	**Group 1:** 61 patients **Group 2:** 36 patients	Nutritional status	Anthropometric indicators; serum albumin level; mineral and vitamin depletion	Oral candidiasis was significantly associated with lower energy and protein intake, reduced leg circumference and lower levels of nutritional proteins, which were related to malnutrition. Patients with oral candidiasis also showed higher rates of zinc and vitamin C deficiency.
**3. Studies without group categorisation**								
Chen 2006	Cross‐sectional	Hospitalised older adults	75.2	NA	114 patients	GOHAI; number of teeth	MNA	GOHAI and number of remaining teeth were not associated with malnutrition.
de Medeiros 2020	Cross‐sectional	Institutionalised older adults	77.7 ± 9.1	NA	344 patients	Masticatory performance; swallowing threshold	MNA‐SF	No correlation between masticatory and swallowing function with MNA‐SF score was observed, but it showed a negative impact on their OHRQoL.
Dormenval 1998	Cross‐sectional	Hospitalised older adults	82.5 ± 4.0	NA	99 patients	USFR; SSFR; dry mouth; loss of appetite	MUAC; triceps skinfold thickness; serum albumin level; BMI	Patients who experience dry mouth or had low saliva production were more likely to suffer from poor nutritional status and overall weakened health. These oral issues appeared to be linked to a reduced appetite and difficulty maintaining adequate nutrition.
Keller 2017	Cross‐sectional	Institutionalised older adults	86.3 ± 7.8	NA	639 patients	Number of teeth; oral health status likely to affect food intake; urgent dental care required	Energy and protein intake	Residents who had poor oral health status likely to affect food intake were associated with lower energy and protein intake.
Kotronia 2020	Prosepctive cohort	Older adults in British Regional Heart Study (BRHS) and the Health, Ageing and Body Composition study (HABC)	78.8 (BRHS) and 74.7 (HABC)	NA	2147 patients (BRHS) and 2998 patients (HABC)	Tooth loss; periodontal disease; self‐rated oral health; dry mouth; cumulated oral health problems	Diet quality; energy intake; energy content from saturated fat; fruit and vegetable intake	BRHS participants with low‐quality diet were significantly related with a higher risk of tooth loss and cumulated oral health problems.
Leung 2016	Cross‐sectional	Community‐dwelling older adults	79.6 ± 7.4	NA	3422 patients	Chewing problems; dry mouth	The presence of any of these five conditions including, unintended weight loss of 5% or more in the last 30 days or 10% or more in the last 180 days, decrease in food eaten, insufficient fluid, cancer and serve malnutrition	Chewing problems and dry mouth were significantly associated with poor nutritional status.
Liang 2020	Cross‐sectional	Older adults living in health and culture village	79	NA	166 patients	Poor chewing ability; GOHAI	MNA	Poor nutritional status was not significantly associated with reduced chewing ability and OHRQoL.
Mojon 1999	Cross‐sectional	Frail older adults with dependency and semi‐dependency	85.0 ± 6.9	NA	324 patients	Oral functional status (fewer than 6 occluding pairs, more than 3 retained roots, or at least one vertical mobility tooth)	BMI; serum albumin level	Compromised oral functional status was associated with BMI, but not with serum albumin levels.
Mudge 2011	Prospective cohort (7‐day follow‐up)	Hospitalised older adults	81	NA	126 patients	Dysphagia	REE	The association between dysphagia and inadequate energy intake was weak.
Ohta 2022	Cross‐sectional	Hospitalised older adults	82.5 ± 7.0	NA	60 patients	Oral hypofunction	MNA‐SF; NRS; GLIM; serum albumin level	Oral hypofunction correlated with risk of malnutrition graded by MNA‐SF and NRS, but not by GLIM and serum albumin level.
Österberg 2002	Cross‐sectional	Community‐dwelling older adults aged 80 years	80	NA	160 patients	Number of teeth; maximum bite force	Diet history; nutritional biomarker	Bite force and masticatory function had weak effects on dietary selection and intake.
Ramage‐Morin and Garriguet 2013	Cross‐sectional	Community‐dwelling older adults	77	NA	15,669 patients	Oral health status	Senior in the community risk evaluation for eating and nutrition II	Women with poor oral health had significantly higher odds of nutritional risk.
Ritchie 1997	Cross‐sectional	Urban homebound older adults	78.0 ± 1.1	NA	49 patients	Denture wearing status; oral health problems e.g., chewing difficulties	BMI; serum albumin level	Chewing problems and inadequate dentures were significantly associated with lower BMI, indicating undernutrition, whereas only inadequate dentures were significantly associated with lower serum albumin levels.
Salmi 2022	Cross‐sectional	Institutionalised frail older adults	84.5 ± 5.4	NA	225 patients	Chewing and swallowing problems; poor appetite; oral health related eating problems	MNA	Risk of malnutrition was significantly associated with problems with chewing, swallowing, poor appetite and oral health‐related eating problems.
Schmalz 2021	Cross‐sectional	Hospitalised older adults	84.2 ± 7.8	NA	151 patients	Oral health status	MNA	Regression analysis demonstrated that MNA score was significantly influenced by DMF‐T, root caries and OHIP‐G14.
Shen 2023	Cross‐sectional	Community‐dwelling older adults	87.9 ± 11.5	NA	54,796 patients	Dentition status	GNRI	Patients with fewer than 20 teeth were not significantly associated with malnutrition status. Inadequate dentition was associated with higher odds of sugar and sweets intake.
Soini 2003	Cross‐sectional	Frail older adults receiving home care services	83.7 ± 4.4	NA	51 patients	Dentition status; oral problems	MNA	Only dry mouth was associated with MNA score and functional natural dentition was associated with BMI.
Solemdal 2012	Cross‐sectional	Acutely hospitalised older adults	83.2 ± 5.9	NA	138 patients	Number of teeth; posterior occluding pairs; mucosal plaque score	BCM; MNA‐SF	Oral health (indicated by having more teeth, more posterior occluding pairs and better oral hygiene) was not significantly associated with BCM and MNA‐SF.
Syrjälä 2013	Cross‐sectional	Dentate, community‐dwelling older adults aged ≥ 75 years	78.6 ± 3.4–80.4 ± 3.6	NA	157 patients	SSFR and USFR	MNA‐SF	There was a statistically significant association between low SSFR/USFR and the risk of malnutrition.
Wu 2018	Cross‐sectional	Community‐dwelling older adults	75.3 ± 6.7	NA	195 patients	GOHAI	MNA	Tooth loss and untreated caries were significantly determined to be poor OHRQoL. Poor OHRQoL was significantly associated with malnutrition.

Abbreviations: BCM, Body Cell Mass; BMI, Body Mass Index; DMF‐T, Decayed, Missing and Filled Teeth index; DVS, Dietary Variety Score; Ed‐FED, Edinburgh Feeding Questionnaire; EER, Estimated Energy Requirement; FTU(s), Functional Tooth Unit(s); GLIM, Global Leadership Initiative on Malnutrition Criteria; GNRI, Geriatric Nutritional Risk Index; GOHAI, Geriatric Oral Health Assessment Index; MNA, Mini Nutritional Assessment; MNA‐SF, Mini Nutritional Assessment–Short Form; MUAC, Mid‐upper arm circumference; MUST, Malnutrition Universal Screening Tool; NA, Not Available/Not Applicable; NRS, Nutritional Risk Screening; NST, Nutrition Screening Tool; OHAT, Oral Health Assessment Tool; OHIP‐G14, Oral Health Impact Profile–German 14‐item version; OHRQoL, Oral Health‐Related Quality of Life; PFU(s), Posterior Functional Unit(s); REE, Resting Energy Expenditure; ROAG, Revised Oral Assessment Guide; ROAG‐J, Revised Oral Assessment Guide–Japanese version; SMI, Skeletal Muscle Mass Index; SNAQ, Short Nutritional Assessment Questionnaire; SSFR, Stimulated salivary flow rate; TCI, Tongue Coating Index; USFR, Unstimulated salivary flow rate.

#### 3.3.7 Oral Health Interventions

Amagai et al. [83] and Kanazawa et al. [85] conducted randomised controlled trials (RCTs) with similar interventions among community‐dwelling edentulous older adults, providing new complete dentures with simple dietary advice compared to regular denture advice. Both studies found a significant improvement in food intake at the 3‐month follow‐up, but Kanazawa et al. reported no significant improvement at 6 months. Additionally, Amagai et al. found that the intervention did not significantly improve OHRQoL.

Müller et al. [86] compared implant‐supported mandibular overdentures with a conventional reline for existing dentures. Both groups showed decreased BMI, but the intervention group had a smaller change. MNA scores and nutritional biomarkers were not significantly different. Wallace et al. [88] compared removable partial dentures and shortened dental arch therapy (10 occluding pairs with adhesive bridges). Both groups had similar chewing capacity, with only minor effects on nutritional status. These studies suggested that improving dental prosthetics did not directly improve nutritional status.

Wu et al. [89] implemented the Eat Ability Promotion Program (EAPP), combining oral muscle‐strengthening exercises and oral hygiene care, which significantly improved both oral health and nutritional status. Beck et al. [84] combined moderate‐intensity exercise, oral training supplements and professional dental care, resulting in significantly improved nutritional status after 11 weeks of follow‐up. Finally, one non‐RCT study by Nihtilä et al. [87] evaluated a tailored xerostomia and nutrition intervention. Participants with xerostomia received customised self‐management recommendations and individualised dietary plans. The intervention significantly reduced xerostomia.

All included studies are reported descriptively in Table 3.

**TABLE 3:** Summary of included interventional studies.

Author/Year	Study design	Population	Age (years)	Study groups	Number of participants (n)	Follow‐up period	Focus outcomes	Main findings
Amagai 2017	RCT	Community‐dwelling older adults with edentulous	**Group 1:** 75.3 ± 8.2 **Group 2:** 78.6 ± 6.6	**Group 1:** new complete dentures and 20 min simple dietary advice **Group 2:** new complete dentures and 20 min denture care advice	**Group 1:** 35 patients **Group 2:** 35 patients	3 months after final denture adjustment	BDHQ; OHIP‐EDENT‐J	Simple dietary advice after complete denture treatment significantly improved food intake in edentulous individuals, but did not significantly improve OHRQoL.
Beck 2010	RCT	Institutionalised older adults	**Group 1:** 87 (84–90) **Group 2:** 86 (84–87)	**Group 1:** moderate intensity exercise 45–60 min + 150 mL oral training supplement + oral care 1–2 times/week (11 weeks) **Group 2:** regular exercise programme + standard nutritional and oral care	**Group 1:** 62 patients **Group 2:** 59 patients	27 weeks after 11 weeks	Weight; BMI; energy and protein intake; functional abilities	The intervention group maintained social and physical functional abilities better than controls. Weight, BMI and protein intake significantly improved over 11 weeks.
Kanazawa 2019	RCT	Community‐dwelling older adults with edentulous	**Group 1:** 78.6 ± 6.8 **Group 2:** 74.8 ± 8	**Group 1:** new complete dentures insertion and simple dietary advice **Group 2:** new complete dentures insertion and denture care advice	**Group 1:** 30 patients **Group 2:** 29 patients	3 months and 6 months	BDHQ	Participants who received both new complete dentures and dietary advice showed a significantly greater short‐term intake of nutrients compared to controls. These findings indicated temporary improvements in nutrient consumption among older adults with edentulism.
Müller 2013	RCT	Institutionalised or receiving help for daily living older adults aged > 75 years	**Group 1:** 85.0 ± 6.2 **Group 2:** 84.1 ± 5.6	**Group 1:** implant‐supported mandibular overdenture **Group 2**: conventional reline of their lower denture	**Group 1:** 16 patients **Group 2:** 18 patients	3 months	BMI; MNA; blood markers; chewing efficiency; bite force; denture satisfaction; denture stability	Converting lower dentures to implant‐supported overdentures significantly improved denture satisfaction, OHRQoL, bite force and masseter muscle thickness compared with a conventional reline. Although BMI declined in both groups, the reduction was smaller in the intervention group, however this trend was not supported by MNA scores or blood biomarkers.
Nihtilä 2019	Non‐RCT	Institutionalised older adults aged ≥ 75 years who were malnourished	**Group 1:** 83.9 ± 90.2 **Group 2:** 83.7 ± 89.9	**Group 1:** tailored xerostomia and nutritional interventions **Group 2**: no intervention	**Group 1:** 119 patients **Group 2:** 97 patients	6 months	Dry mouth; MNA; daily eating and drinking habits	Dry mouth was reduced by 30% and the prevalence of malnutrition or risk of malnutrition dropped by 61% among participants who received both interventions.
Wallace 2018	RCT	Community‐dwelling older adults	**Group 1:** 79.6 ± 6.5 **Group 2:** 79.7 ± 5.4	**Group 1:** shortened dental arch concept (SDA) using adhesive bridgework **Group 2:** removable partial denture (RPDs)	**Group 1:** 45 patients **Group 2:** 44 patients	6 months	Masticatory performance; MNA; biochemical markers	There was an improvement in masticatory function in those partially dentate rehabilitated with either RPDs or SDA, but masticatory performance itself did not improve the nutritional state.
Wu 2020	RCT	Community‐dwelling older adults	76.1	**Group 1:** Eating Ability Promotion Programme 2 × 4 h/per week for 12 weeks **Group 2:** originally scheduled activities only	**Group 1:** 37 patients **Group 2:** 30 patients	1 month	OHAT; MNA; BMI; SOF; Mini‐Cog	The Eating Ability Promotion Programme significantly improved oral health, nutritional status and reduced frailty compared with controls. These benefits persisted for at least 1 month after the intervention.

Abbreviations: BDHQ, Brief‐type self‐administered Diet History Questionnaire; BMI, Body Mass Index; Mini‐Cog, Short Cognitive Impairment Screening Exam; MNA, Mini Nutritional Assessment; MNA‐SF, Mini Nutritional Assessment–Short Form; OHAT, Oral Health Assessment Tool; OHIP‐EDENT‐J, Japanese version of the Oral Health Impact Profile for Edentulous; OHRQoL, Oral Health‐Related Quality of Life; RCT, Randomised Controlled Trial; SOF, Study of Osteoporotic Fractures.

### 3.4 Synthesis of Results: Meta‐Analyses of the Outcomes

All included studies are reported descriptively in Table 4.

**TABLE 4:** Summary of included studies for meta‐analysis.

Author/Year	Study design	Population	Age (years)	Study groups	Number of participants (*n*)	Focus outcomes	Nutritional evaluation tools	Main findings
Ambrosio‐Palma 2022	Cross‐sectional	Outpatients of geriatric evaluation clinic	81.2 ± 7.5	**Group 1:** without dysphagia **Group 2:** with dysphagia	**Group 1:** 79 patients **Group 2:** 21 patients	Nutritional status	MNA‐SF	Malnutrition and xerostomia were significantly associated with swallowing disorders.
Boulos 2014	Cross‐sectional	Community‐dwelling older adults	75.7 ± 7.1	NA	1200 patients	Chewing problems; edentulism; denture use	MNA	All oral health outcomes were significantly associated with malnutrition.
Chou 2023	Cross‐sectional	Institutionalised older adults	80.4 ± 11.7	**Group 1:** well‐nourished **Group 2:** at risk of malnutrition	**Group 1:** 21 patients **Group 2:** 18 patients	OHAT; EAT‐10; FOIS	MNA‐SF	Poor oral health and poor swallowing function were significantly associated with at risk of malnutrition. OHAT and EAT‐10 scores showed a positive correlation with MNA‐SF.
Furuta 2013	Cross‐sectional	Older adults receiving home care service	84.5 ± 7.9	NA	286 patients	Nutritional status	MNA‐SF	Nutritional status was not different regarding the number of teeth and denture‐wearing status but significantly different when having dysphagia.
Gao 2025	Cross‐sectional	Community‐dwelling older adults	80.0 ± 2.0	**Group 1:** well‐nourished **Group 2:** malnutrition	**Group 1:** 137 patients **Group 2:** 73 patients	Tongue and lip closing pressure; molar occlusal force; chewing function; tastes	MNA‐SF	Reduced tongue pressure and molar occlusal force, taste perception and self‐reported chewing difficulties were significantly associated with malnutrition.
Gil‐Montoya 2013	Cross‐sectional	Institutionalised older adults	82.7 ± 8.2	**Group 1:** well‐nourished **Group 2:** malnutrition/at risk	**Group 1:** 158 patients **Group 2:** 92 patients	Number of teeth and occlusal pairs; OHIP‐14	MNA	OHRQoL and number of occlusal pairs were significantly related to nutritional status rather than number of teeth.
Griep 2000	Cross‐sectional	Institutionalised older adults	83.4 ± 6.6	**Group 1:** well‐nourished **Group 2:** at risk of malnutrition **Group 3:** malnutrition	**Group 1:** 51 patients **Group 2:** 28 patients **Group 3:** 2 patients	Number of teeth; type of denture use	MNA	Number of teeth was not associated with nutritional status.
Jürschik 2010	Cross‐sectional	Older adults receiving different levels of care	77.0 ± 7.2	**Group 1:** malnutrition/at risk **Group 2:** well‐nourished	**Group 1:** 231 patients **Group 2:** 167 patients	Chewing and swallowing difficulties	MNA	Older patients with difficulty chewing and swallowing were significantly associated with unsatisfactory nutrition.
Khoury 2022	Cross‐sectional	Community‐dwelling and institutionalised older adults	94.1 ± 3.0	**Group 1:** well‐nourished **Group 2:** malnutrition/at risk	**Group 1:** 64 patients **Group 2:** 23 patients	DMF‐T; posterior occluding pairs; oral pain; dry mouth; denture use	MNA	After adjusting for various factors, only xerostomia was significantly associated with being malnourished or at risk of malnutrition.
Kikutani 2013	Cross‐sectional	Community‐dwelling frail older adults	78.7 ± 9.0	**Group 1:** well‐nourished **Group 2:** at risk of malnutrition **Group 3:** malnutrition	**Group 1:** 251 patients **Group 2:** 370 patients **Group 3:** 95 patients	Natural dentition and denture‐wearing status; swallowing problems	MNA	The loss of occlusion in natural dentition was associated with risk of malnutrition, while swallowing problems were associated with malnutrition.
van Kuijk 2021	Cross‐sectional	Institutionalised older adults	83.5	NA	987 patients	Nutritional status	MNA‐SF	Number of teeth and edentulism were not significantly associated with nutritional status.
Meguro 2022	Cross‐sectional	Older adults requiring long‐term care	86.6 ± 6.9	**Group 1:** well‐nourished/at risk **Group 2:** malnutrition	**Group 1:** 265 patients **Group 2:** 57 patients	Dentition; lip‐closure ability; ODK; dry mouth; TCI	MNA‐SF	Having fewer than 20 teeth with dentures was significantly associated with lower risk of malnutrition, whereas poor lip‐closure ability was significantly associated with malnutrition.
Soini 2006	Cross‐sectional	Institutionalised older adults	81–83	**Group 1:** nursing home **Group 2:** long‐term care	**Group 1:** 2036 patients **Group 2:** 1052 patients	Dentition type; chewing/swallowing difficulties; dry mouth; pain in mouth	MNA	Residents who were totally edentulous without prosthesis and those with multiple oral health problems were more likely to be malnourished or at risk of malnutrition.
Won 2024	Prospective cohort (2‐year follow‐up)	Community‐dwelling older adults	76.5 ± 3.9	**Group 1:** without masticatory difficulties **Group 2:** with masticatory difficulties	**Group 1:** 1856 patients **Group 2:** 1154 patients	Nutritional status	MNA	Patients with baseline masticatory difficulties were associated with the incidence of malnutrition.
Wu 2024	Cross‐sectional	Community‐dwelling older adults	81.4 ± 4.3	**Group 1:** well‐nourished **Group 2:** malnutrition/at risk	**Group 1:** 164 patients **Group 2:** 112 patients	Number of teeth; denture type; oral problems (e.g., xerostomia); oral function (e.g., dysphagia)	MNA‐SF	Poor self‐rated chewing ability, xerostomia, bite force, tongue and lip sealing pressure and dysphagia were significantly higher in the malnourished group.
Yodogawa 2022	Cross‐sectional	Community‐dwelling older adults	76.3 ± 7.3	**Group 1:** well‐nourished **Group 2:** malnutrition/at risk	**Group 1:** 173 patients **Group 2:** 65 patients	OHIP‐14; ODK; number of teeth	MNA	ODK and OHIP‐14 scores and number of teeth were significantly higher in well‐nourished group.

Abbreviations: DMF‐T, Decayed, Missing and Filled Teeth; EAT‐10, Eating Assessment Tool; FOIS, Functional Oral Intake Scale; GOHAI, Geriatric Oral Health Assessment Index; MNA, Mini Nutritional Assessment; MNA‐SF, Mini Nutritional Assessment–Short Form; NA, Not Available/Not Applicable; ODK, Oral Diadochokinesis; OHAT, Oral Health Assessment Tool; OHIP‐14, Oral Health Impact Profile‐14; OHRQoL, Oral Health–Related Quality of Life; TCI, Tongue Coating Index.

#### 3.4.1 Association Between Nutritional Status and Number of Teeth and Removable Dental Prosthesis

**FIGURE 2:**
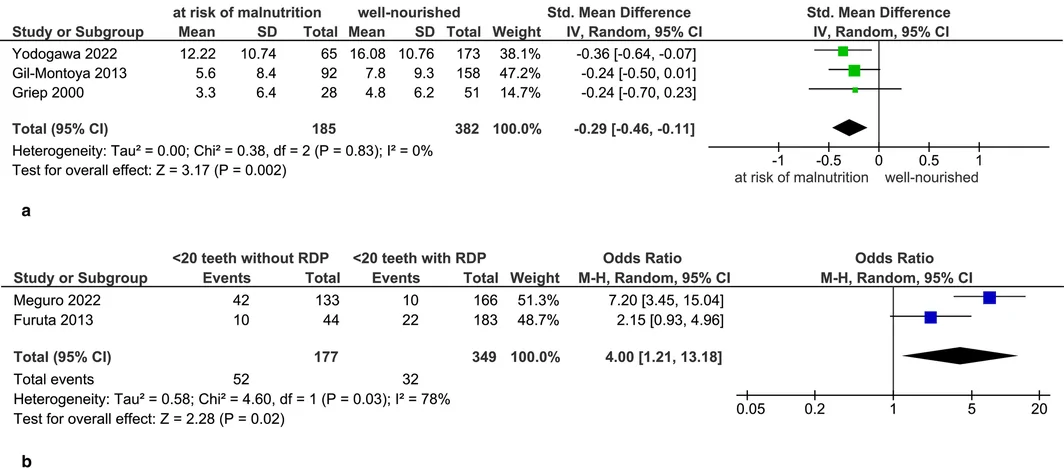
Forest plots comparing the number of teeth between those at risk of malnutrition and well‐nourished older adults (a) and the odds of malnutrition compared between older adults who had less than 20 teeth with and without RDP (b). Chi^2^, Chi‐squared; CI, confidence interval; *I* ^2^, *i*‐squared; IV, inverse variance; M‐H, Mantel‐Haenzel; RDP, Removable dental prosthesis; SD, standard deviation; Std. mean difference, standardised mean difference; Tau^2^, Tau‐squared. [Colour figure can be viewed at wileyonlinelibrary.com]

Three studies reported the number of teeth between well‐nourished and at risk of malnutrition group were included in the analysis [94, 95, 105]. Well‐nourished participants had an average of 4.8–16.08 teeth, while those at risk had 3.3–12.22 teeth. The meta‐analysis found that those at risk had significantly fewer teeth (SMD = –0.29; 95% CI: −0.46, −0.11; *p* = 0.002), with no evidence of heterogeneity beyond that expected by chance (*I* ^2^ = 0%) (Figure 2a).

A total of 2 studies compared older adults who had less than 20 teeth with removable dental protheses and those without removable dental protheses were included in the analysis [91, 93]. The meta‐analysis revealed that those without dentures had significantly higher odds of malnutrition (OR = 4.00; 95% CI: 1.21, 13.18; *p* = 0.02), but there was with high heterogeneity (*I* ^2^ = 78%) (Figure 2b).

#### 3.4.2 Association Between Nutritional Status and Chewing Problems

**FIGURE 3:**
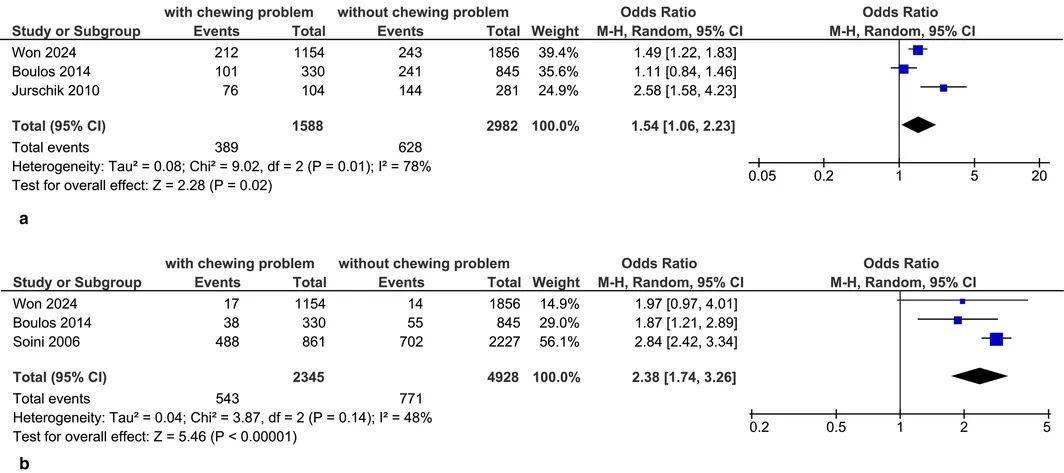
Forest plots of the odds of malnutrition risk (a) and malnutrition (b) compared between older adults with and without self‐reported subjective chewing problems. Chi^2^, Chi‐squared; CI, confidence interval; *I* ^2^, *i*‐squared; M‐H, Mantel‐Haenszel; Tau^2^, Tau‐squared. [Colour figure can be viewed at wileyonlinelibrary.com]

A total of 4 studies compared older adults with and without chewing problems in relation to malnutrition/at risk that were included in the analysis. Won et al. [103] defined chewing problems using a five‐point scale for the question ‘Do you experience discomfort when chewing food due to problems with your teeth, dentures, gums or others?’, while Boulos et al. [92], Jürschick et al. [98] and Soini et al. [96] defined it using a dichotomous question on chewing problems. The meta‐analysis revealed that those with chewing problems had a non‐significant association with the risk of malnutrition (OR = 1.18; 95% CI: 0.59, 2.36; *p* = 0.64). The analysis revealed high heterogeneity (*I* ^2^ = 97%). Sensitivity analysis was performed by excluding data from Soini et al. [96] which created the highest heterogeneity, resulting in significantly higher odds of being at risk of malnutrition (OR = 1.54; 95% CI: 1.06, 2.23; *p* = 0.02). Nevertheless, the heterogeneity across the studies was still high (*I* ^2^ = 78%) (Figure 3a). Three studies from above had sufficient information, allowing a pooled analysis that showed those with chewing problems were also at significantly higher odds of malnutrition (OR = 2.38; 95% CI: 1.74, 3.26; *p* < 0.0001), with moderate heterogeneity (*I* ^2^ = 48%) (Figure 3b).

#### 3.4.3 Association Between Nutritional Status and Swallowing Problems

**FIGURE 4:**
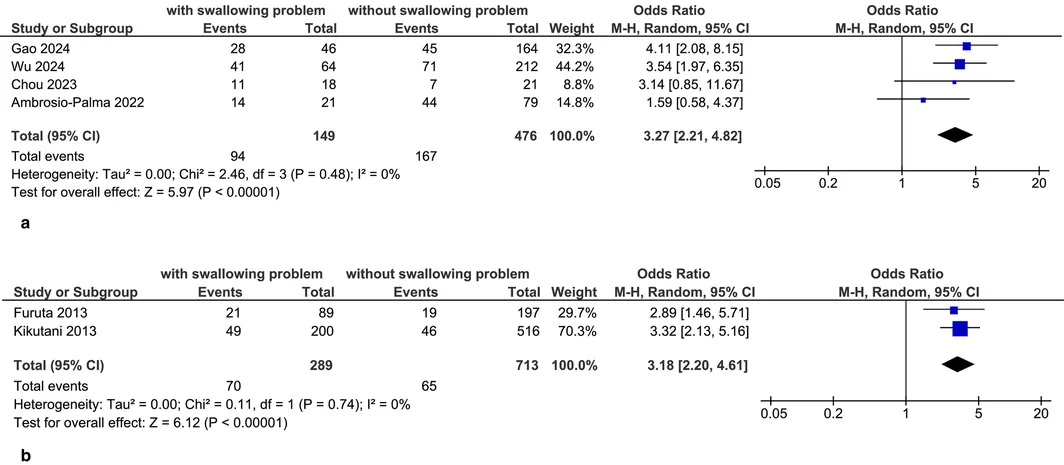
Forest plots of the odds of malnutrition risk compared between older adults with and without self‐reported subjective (EAT‐10) swallowing problem (a) and malnutrition compared between older adults with and without objective (Cervical auscultation) swallowing problem (b). Chi^2^, Chi‐squared; CI, confidence interval; *I* ^2^, *i*‐squared; M‐H, Mantel‐Haenszel; Tau^2^, Tau‐squared. [Colour figure can be viewed at wileyonlinelibrary.com]

A total of 4 studies reported the effect of swallowing problems measured by the Eating Assessment Tool (EAT‐10) that were included in the analysis [90, 97, 101, 104]. The meta‐analysis showed significantly higher odds of the risk of malnutrition among those with subjective swallowing problems (OR = 3.27; 95% CI: 2.21, 4.82; *p* < 0.0001) with no evidence of heterogeneity beyond that expected by chance (*I* ^2^ = 0%) (Figure 4a). The study from Soini et al. [96] was not included in the analysis because of the use of a non‐validated questionnaire to detect swallowing problems.

Objective measurement of swallowing problems using the cervical auscultation method was conducted in 2 studies [93, 107]. The meta‐analysis showed a significantly higher odds of malnutrition (OR = 3.18; 95% CI: 2.20, 4.61; *p* < 0.0001) (Figure 4b). There was no observed heterogeneity beyond that expected by chance (*I* ^2^ = 0%). Studies from Poisson et al. [61] and Forcano et al. [37] using a water test and a volume viscosity swallowing test, respectively, were excluded from the analysis due to methodological differences.

#### 3.4.4 Association Between Nutritional Status and Dry Mouth

**FIGURE 5:**
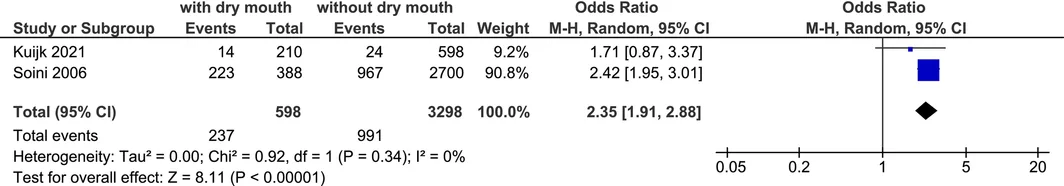
Forest plot of the odds of malnutrition compared between older adults with and without dry mouth. Chi^2^, Chi‐squared; CI, confidence interval; *I* ^2^, *i*‐squared; M‐H, Mantel‐Haenszel; Tau^2^, Tau‐squared. [Colour figure can be viewed at wileyonlinelibrary.com]

Subjective symptoms of dry mouth were measured by a dichotomous questionnaire in three studies from Gao et al. [97], Kuijk et al. [102] and Soini et al. [96], while Khoury et al. [99] used the Xerostomia Inventory (XI) questionnaire. The meta‐analysis from those studies using similar measurements was omitted due to a non‐significant association with being at risk of malnutrition (OR = 1.34; 95% CI: 0.62, 2.89; *p* = 0.46) with high heterogeneity across the studies (*I* ^2^ = 93%). Conversely, studies by Kuijk et al. [102] and Soini et al. [96] allowed the analysis of malnutrition, which showed higher odds of malnutrition in older adults with dry mouth (OR = 2.35; 95% CI: 1.91, 2.88; *p* < 0.0001) with no heterogeneity observed beyond what would be expected by chance (*I* ^2^ = 0%) (Figure 5). Additionally, studies from Meguro et al. [91] and El Helou et al. [106] were not included in the analysis due to an inconsistent methodology using inspection of the oral cavity for dryness.

#### 3.4.5 Association Between Nutritional Status and Oral Pain

**FIGURE 6:**
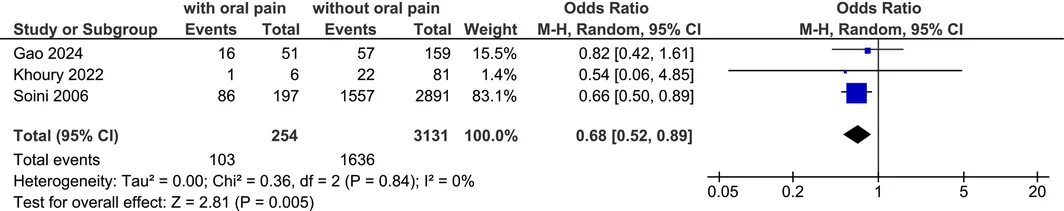
Forest plot of the odds of malnutrition risk compared between older adults with and without oral pain. Chi^2^, Chi‐squared; CI, confidence interval; *I* ^2^, *i*‐squared; M‐H, Mantel‐Haenszel; Tau^2^, Tau‐squared. [Colour figure can be viewed at wileyonlinelibrary.com]

A total of 3 studies were included in the analysis. The studies from Gao et al. [97], Khoury et al. [99] and Soini et al.^96^ assessed subjective oral pain by a dichotomous questionnaire. The meta‐analysis indicated that older adults with oral pain had significantly lower odds of being at risk of malnutrition (OR = 0.68; 95% CI: 0.52, 0.89; *p* = 0.005), with no evidence of heterogeneity beyond that expected by chance (*I* ^2^ = 0%) (Figure 6).

### 3.5 Risk of Bias and Quality Assessment of the Included Studies

**FIGURE 7:**
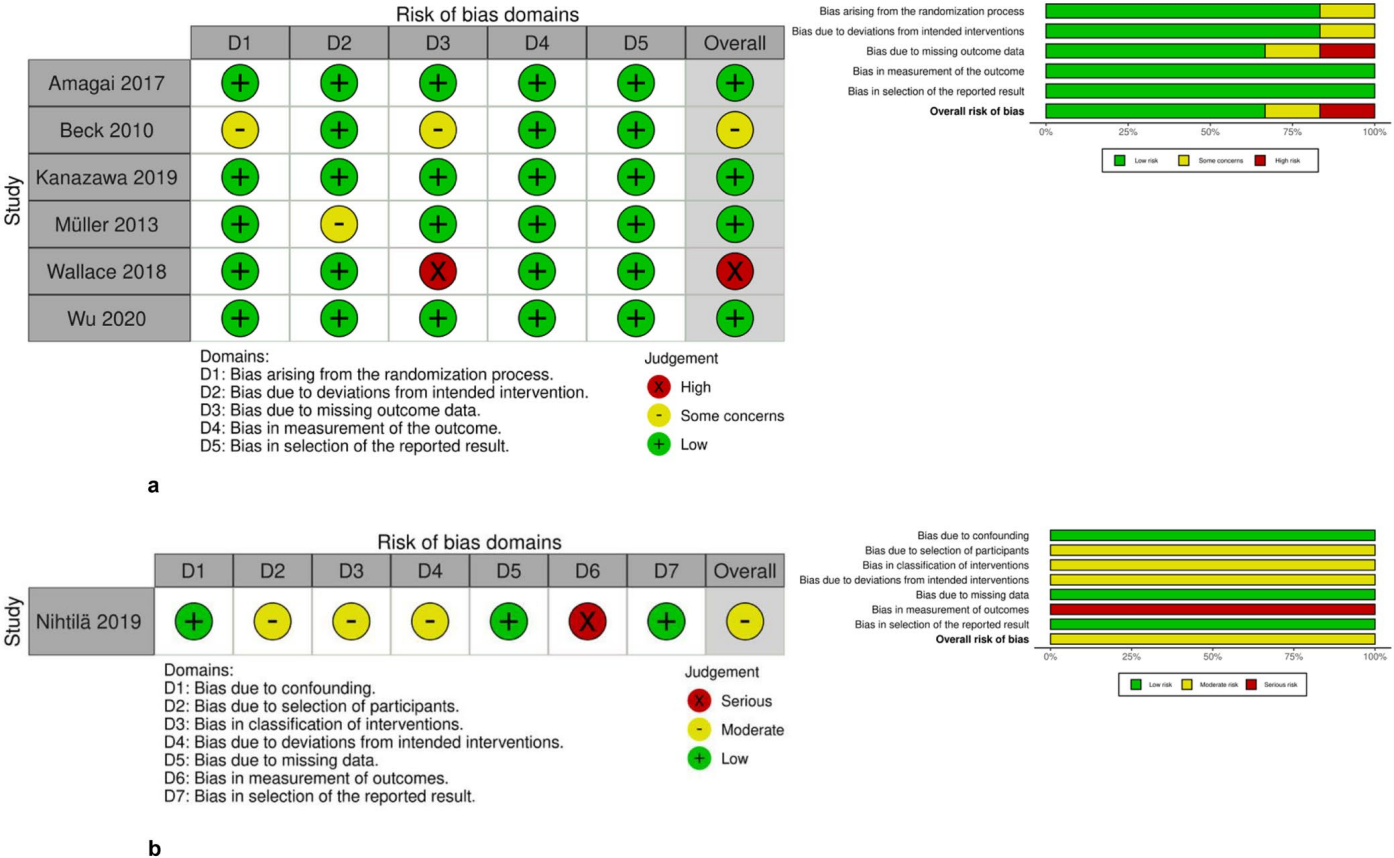
Traffic light plot and summary plot of the risk of bias assessment for included randomised controlled trials, evaluated using the Cochrane tool for the assessment of the risk of bias (RoB 2) (a) and non‐randomised controlled trials, evaluated using the non‐randomised studies of interventions (ROBINS‐I) (b). [Colour figure can be viewed at wileyonlinelibrary.com]

The risk of bias for the included studies in the meta‐analysis was assessed as ‘low’ (9 studies) and ‘moderate’ (7 studies). Detailed assessments can be found in Table 5. The remaining studies used in the descriptive analysis of association were assessed as mainly ‘low’ (46 out of 61 studies). Detailed assessments can be found in Table 6. Similarly, Intervention studies were assessed as mainly ‘low’ (4 out of 7 studies). Detailed assessments can be found in Figure 7a,b.

**TABLE 5:** Risk of bias of included studies for meta‐analysis using Newcastle‐Ottawa scale.

First author (Year)	Selection					Comparability		Outcome			
	Representation of exposed cohort	Sample size	Non‐respondents	Ascertainment of exposure (risk factor)		The subjects in different outcome groups are comparable, based on the study design or analysis. Confounding factors are controlled		Assessment of outcome		Statistical test	Total (max 10) 0–3 high 4–6 moderate 7–10 low
Ambrosio‐Palma (2022)		*		*	*	*		*	*	*	7
Boulos (2014)	*	*	*	*	*	*	*	*	*	*	10
Chou (2023)		*		*		*		*	*	*	6
Furuta (2012)	*	*		*		*	*	*	*	*	8
Gao (2024)	*	*	*	*	*	*	*	*	*	*	10
Gil‐Montoya (2013)	*	*		*		*		*		*	6
Griep (2000)	*	*		*		*		*		*	6
Jürschik (2010)	*	*		*		*		*	*	*	7
Khoury (2022)	*	*	*	*		*		*		*	7
Kikutani (2013)	*	*		*	*	*		*	*	*	8
Kuijk (2021)	*	*		*		*		*	*	*	6
Meguro (2022)	*	*		*		*		*		*	6
Soini (2006)	*	*	*	*		*		*		*	8
Won (2024)	*	*		*	*	*		*	*	*	8
Wu (2024)	*	*		*		*		*		*	6
Yodogawa (2022)	*	*		*		*		*		*	6

*indicates that a point (score) is awarded for that specific criterion.

**TABLE 6:** Risk of bias of included studies for descriptive analysis using Newcastle‐Ottawa scale.

First author (Year)	Selection					Comparability		Outcome			
	Representation of exposed cohort	Sample size	Non‐respondents	Ascertainment of exposure (risk factor)		The subjects in different outcome groups are comparable, based on the study design or analysis. Confounding factors are controlled		Assessment of outcome		Statistical test	Total (max 10) 0–3 high 4–6 moderate 7–10 low
Bakker (2018)	*	*	*	*	*	*	*	*	*	*	10
Chiesi (2019)		*	*	*	*	*	*	*	*	*	9
Dewake (2017)		*		*		*		*	*	*	6
El Helou (2014)		*	*	*		*		*		*	6
El Osta (2022)		*		*	*	*	*	*	*	*	8
Feldblum (2007)		*	*	*	*	*	*	*	*	*	9
Forcano‐Sanjuan (2018)		*		*	*	*		*	*		6
Fukuyama (2024)	*	*		*	*	*	*	*	*	*	9
Holst (2013)		*	*	*		*		*		*	6
Hua (2022)		*		*	*	*	*	*	*	*	8
Izumi (2020)		*	*			*		*		*	5
Kucuk and Kapucu (2017)		*	*	*	*	*	*	*	*	*	9
Lindmark (2017)	*	*	*	*	*	*		*	*	*	9
Lopez‐Jornet (2013)	*	*	*	*	*	*	*	*	*	*	10
Nishio (2024)	*	*	*	*	*	*	*	*	*	*	10
Nykänen (2013)	*	*	*	*	*	*	*	*	*	*	10
Soini (2005)		*	*	*	*	*		*	*	*	8
Stratidaki (2024)	*	*	*	*	*	*	*	*	*	*	10
Van Der Pols‐Vijlbrief (2014)	*	*	*	*	*	*	*	*	*	*	10
Zenthöfer (2015)		*		*	*	*	*	*			6
Han and Kim (2016)				*		*	*	*			4
Julkunen (2024)		*		*		*		*		*	5
Krzyminska‐Siemaszko (2016)		*	*	*	*	*	*	*	*	*	9
Lamy (1998)		*	*	*	*	*	*	*	*	*	9
Papas (1998)	*	*		*		*		*		*	6
Saarela (2014)		*	*	*		*		*	*	*	7
Saarela (2016)		*		*	*	*	*	*	*	*	8
Sadamori (2012)		*	*	*		*	*	*	*	*	8
Chew (2023)		*		*	*	*	*	*	*	*	8
Galesi‐Pacheco (2021)		*		*	*	*	*	*	*	*	8
Saarela (2019)		*	*	*	*	*	*	*			7
Shiraishi (2017)		*		*	*	*	*	*	*	*	7
Leung (2016)	*	*		*	*	*	*	*	*	*	8
Mann (2013)	*	*		*	*	*	*	*	*	*	9
Wakabayashi (2018)		*		*	*	*	*	*	*	*	8
Iwasaki (2020)	*	*	*	*	*	*		*	*	*	9
Iwasaki (2021)	*	*		*		*	*	*	*	*	8
Nakagawa (2024)	*	*		*		*		*	*		6
Iwasaki (2016)	*	*		*		*	*	*	*	*	8
Leung (2016)	*	*	*	*		*	*	*	*	*	9
Paillaud (2004)		*		*		*	*	*	*	*	7
Poisson (2014)		*		*		*	*	*	*	*	7
Chen (2006)		*		*	*	*	*	*	*	*	8
Davino de Medeiros (2020)		*	*	*		*	*	*	*	*	8
Dormenval (1998)		*		*		*		*	*	*	6
Keller (2017)		*	*	*	*	*		*	*	*	9
Kotronia (2020)	*	*		*		*		*		*	6
Liang (2020)	*	*		*	*	*	*	*	*	*	9
Mojon (1999)		*		*		*		*		*	5
Mudge (2011)		*		*		*		*		*	5
Ohta (2022)		*	*	*	*	*		*	*	*	8
Österberg (2008)	*	*		*	*	*		*	*	*	8
Ramage‐Morin and Garriguet (2013)	*	*		*	*	*		*		*	7
Ritchie (1997)		*	*	*	*	*		*	*	*	8
Salmi (2022)		*	*	*	*	*		*	*	*	8
Schmalz (2021)		*	*	*	*	*	*	*	*	*	9
Shen (2023)	*	*	*	*		*	*	*	*	*	9
Soini (2003)		*	*	*	*	*		*		*	7
Solemdal (2012)		*		*	*	*		*	*		6
Syrjälä (2013)		*		*	*	*	*	*		*	7
Wu (2018)		*	*	*	*	*	*	*		*	8

*indicates that a point (score) is awarded for that specific criterion.

## 4 Discussion

This systematic review evaluated whether nutritional status was associated with oral health factors and vice versa, particularly in older adults aged 75 years or older. The average number of teeth was significantly lower in the group at risk of malnutrition. However, in all three studies included in the pooled analysis, both comparison groups had fewer than 20 teeth, suggesting that differences might be more pronounced among patients with very few remaining teeth. Importantly, information about denture‐wearing status was often unclear across all studies. Descriptive analysis mainly found no association between denture use and nutritional status, although the specific type of dentures used and the dentition status of non‐wearers were not always specified. Interestingly, some studies indicated that patients who needed dentures but did not wear them were more likely to experience chewing difficulties that affected their nutrition. In our pooled analysis, two studies showed a significant association between not wearing removable dentures and an increased risk of malnutrition among patients with fewer than 20 teeth, but there was high heterogeneity between studies. More detailed comparisons between denture wearers and non‐wearers, particularly among edentulous and partially edentulous individuals, are needed and should be clearly reported in future research.

Chewing problems consistently showed a strong association with poor nutritional status in both descriptive and pooled analyses, as confirmed by significantly higher odds of malnutrition and risk of malnutrition in the pooled effect. This finding aligns with Hussein et al. [6] and highlights the significant impact of self‐perceived chewing difficulty on nutritional status. Therefore, a reduced number of teeth, a need for dentures, and chewing problems can all negatively affect nutritional status. This suggests that, beyond simply increasing the number of teeth, nutritional status may be improved by interventions that enhance chewing efficiency [108].

Swallowing problems also showed consistent associations with nutritional status in both descriptive and pooled analyses, but dry mouth demonstrated a clear association only in the pooled results. While these factors were not examined in previous systematic reviews, their influence is biologically plausible. Chewing initiates the oral phase of digestion; saliva is essential for forming the food bolus, and swallowing ensures its transport into the gastrointestinal tract. Chewing difficulties can impair the swallowing process and further impact digestive efficiency [109]. Similarly, salivary hypofunction can negatively affect both chewing and swallowing abilities [110, 111, 112]. Moreover, xerostomia patients are more likely to avoid certain foods, further impacting nutrtion [113]. The significant associations of oral health outcomes including chewing, swallowing and xerostomia highlight the bi‐directional relationship with malnutrition in older adults [66]. Importantly, the European Society for Clinical Nutrition and Metabolism (ESPEN) has stated that masticatory and swallowing problems are potential causes of malnutrition. Furthermore, ESPEN recommends reviewing medications that may cause xerostomia, as this can negatively affect nutritional status [114].

Our descriptive findings also indicate that oral interventions such as denture improvement, whether implant‐retained overdentures or the shortened dental arch concept compared to conventional removable dentures, did not significantly improve nutritional status [86, 88, 115]. These interventions primarily address the mechanical ability to fill gaps left by missing teeth, and while they may alleviate chewing difficulties, optimal results may require high‐quality prostheses combined with functional rehabilitation (e.g., masticatory training or chewing exercises) [107, 108, 116] or changes in eating habits (e.g., dietary advice) [117]. Moreover, improving salivary flow can further support both chewing and swallowing processes. However, evidence supporting the utilisation of saliva substitutes to facilitate food bolus formation and lubricate the oropharyngeal mucosa during swallowing remains limited [118]. Swallowing rehabilitation not only facilitates safe oral intake but may also enhance nutritional status and reduce the risk of aspiration pneumonia [119]. Optimally, evidence suggests that combined interventions targeting both oral and general health may be more effective in addressing malnutrition in older adults [120].

Conversely, oral pain was not significantly associated with the risk of malnutrition. However, the dichotomous questionnaire used in the analysis lacked specificity on the cause of pain, so this finding should be interpreted cautiously and cannot be generalised to any pain in the mouth. The study found that caries treatment and prosthetic needs in older adults were associated with oral pain and eating problems [121]. Additionally, non‐odontogenic oral pain was correlated with other general health issues of older adults such as depressive symptoms and excessive daytime sleepiness, which might indirectly impact nutritional status [122]. However, the association between dry mouth or oral pain and nutritional status should be interpreted with caution. These symptoms may not directly lead to malnutrition but may cooccur as a result of shared underlying factors such as polypharmacy, systemic disease or psychosocial distress. Therefore, such associations may reflect spurious correlations, which are acknowledged in the present review.

### 4.1 Limitations of the Review

Each pooled analysis combined data from different populations, including community‐dwelling older adults (7 studies), institutionalised older adults (5 studies), care‐dependent older adults (3 studies) and geriatric outpatients (1 study). This approach was taken due to the limited number of studies available for each outcome and to increase statistical power for more robust results. A previous meta‐analysis by Toniazzo et al. [7], which used subgroup analyses of institutionalised and non‐institutionalised populations, reported similar findings regarding the impact of edentulism and the number of prostheses on nutritional status. Nevertheless, these results cannot be generalised to all older adults and should be interpreted with caution.

Although more than half of the studies included in the meta‐analysis had a low risk of bias, seven studies were rated as having a moderate risk, most commonly due to the use of non‐validated tools for assessing oral health outcomes. In addition, several studies reported variables such as oral health, oral hygiene and oral frailty/hypofunction; however, variations in measurement tools or insufficient reporting prevented their inclusion in the meta‐analysis. Inconsistent definitions of malnutrition and the use of different nutritional assessment tools further limited the ability to include them in the analysis. A further limitation was that some potentially eligible studies did not report the mean age of participants and attempts to contact the corresponding authors for clarification were unsuccessful, leading to the exclusion of potentially includable papers.

Importantly, the lack of longitudinal studies necessitated reliance on cross‐sectional designs, which can demonstrate associations but cannot allow for conclusions about causal relationships or indicate the role of oral health as a risk factor for malnutrition and vice versa.

### 4.2 Implication for Research

Future research should prioritise longitudinal studies to observe the progression of nutritional status in relation to oral health over time. Standardised and validated tools should be used for assessing oral health, rather than relying solely on interviews or self‐reports. For nutrition, validated tools such as the MNA and MNA‐SF should be employed to facilitate pooled analyses. However, there is a fundamental lack of consensus on diagnostic criteria for their application in clinical settings. Recently, GLIM established a global consensus for diagnosing and assessing malnutrition in adults, demonstrating good diagnostic performance with high sensitivity and specificity [123]. They suggested a two‐step process, starting with screening for malnutrition with tools like MNA‐SF or NRS‐2002, then an assessment for diagnosis, and finally grading the severity of malnutrition [124]. Additionally, the assessment incorporates phenotypic criteria such as weight loss, low BMI or reduced muscle mass (measured by technical approaches or alternative methods, e.g., calf circumference) [125].

In addition to undernutrition, obesity, which was not considered in this review question, has a high prevalence among older adults [126]. Previous studies have demonstrated its association with the number of teeth and edentulism (whether wearing complete dentures, only an upper denture, or no denture at all) [127, 128, 129]. Furthermore, older adults with inadequate dentition tend to have higher sugar and sweets intake, which might worsen obesity [70]. Future systematic reviews should emphasise this condition to provide a more comprehensive understanding of the relationship between nutritional status and oral health more thoroughly. Furthermore, interventional studies specifically targeting individuals aged 75 and older are needed to evaluate the impact of oral health interventions, both alone and in combination with general health improvement strategies.

## 5 Conclusions

This systematic review and meta‐analysis conclude that fewer teeth, existing chewing and swallowing problems, dry mouth and the absence of removable dentures where needed, were associated with malnutrition in care‐dependent older adults. Although descriptive analyses suggest that poor oral health, inadequate oral hygiene and oral frailty or hypofunction may be linked to poorer nutritional outcomes, insufficient information precluded the pooling of this evidence. The current lack of longitudinal studies and proof of causality for malnutrition affecting oral health outcomes underscores the need for further research to better clarify the complex relationship between oral health and nutrition in older populations.

## Author Contributions

K.S., I.W. and M.S. were responsible for writing – review and editing, writing – original draft, supervision, methodology, investigation, formal analysis, data curation, conceptualisation. G.M., P.P. and F.M. were writing – review and editing, supervision, methodology, investigation, data curation, conceptualisation. S.M., C.L. and J.W. were writing – review and editing, supervision, methodology, investigation, data curation. All authors reviewed and approved the final version of the manuscript.

## Funding

The authors have nothing to report.

## Ethics Statement

The authors have nothing to report.

## Consent

All authors confirm their participation in the study leading to this manuscript and consent to its submission to be considered for publication.

## Conflicts of Interest

The authors declare no conflicts of interest.

## Supporting information


**Data S1:** PRISMA Checklist.

**Data S2:** Excluded studies with reasons.

## Data Availability

Data sharing not applicable to this article as no datasets were generated or analysed during the current study.
