# Supplementary material for: The Impact of Nutritional Status on Oral Health Outcomes and Management in Older Adults: A Systematic Review and Meta‐Analysis
Source: Gerodontology. 2025 Dec 2;43(3):420–55. doi: 10.1111/ger.70030 (PMC13498936; doi:10.1111/ger.70030)
Supplement: Supplementary file 2 [file GER-43-420-s002.docx]

**Supplementary material 1:** Excluded studies with reasons

| **Exclusion reasons** | **Authors** | **Year** | **Title** | **DOI** |
| --- | --- | --- | --- | --- |
| Ineligible age | Spanish Geriatric Oral Health Research Group. | 2001 | Oral health issues of Spanish adults aged 65 and over. The Spanish Geriatric Oral Health Research Group | https://pubmed.ncbi.nlm.nih.gov/11561883/ |
| Ineligible age | Kossioni and Bellou | 2011 | Eating habits in older people in Greece: the role of age, dental status and chewing difficulties | doi:10.1016/j.archger.2010.03.017 |
| Ineligible age | Li | 2021 | Dietary inflammatory potential is associated with poor periodontal health: A population-based study | doi:10.1111/jcpe.13472 |
| Ineligible age | Sheiham | 1999 | The impact of oral health on stated ability to eat certain foods; findings from the National Diet and Nutrition Survey of Older People in Great Britain | doi:10.1111/j.1741-2358.1999.00011.x |
| Ineligible age | Yoshihara | 2003 | Association between serum albumin and root caries in community-dwelling older adults | doi:10.1177/154405910308200313 |
| protocol | protocol | 2016 | Do moderate protein, high carbohydrate predominantly plant-based diets assist in appetite control and improve short-term health outcomes in individuals aged 65-75 years? |  |
| protocol | protocol | 2020 | The effect of green-shell mussel on joint health in elderly women |  |
| protocol | protocol | 2021 | The effect of pine bark extract dietary supplement on physical health outcomes in healthy older adults |  |
| Ineligible age | Mesas | 2010 | Oral health status and nutritional deficit in noninstitutionalized older adults in Londrina, Brazil | doi:10.1590/s1415-790x2010000300007 |
| review | Nizel | 1976 | Role of nutrition in the oral health of the aging patient |  |
| review | Aida | 2023 | Epidemiology of oral health on nutrition and food intake | doi:10.1159/000530786 LK - |
| Ineligible age | Al-Zahrani | 2003 | Obesity and periodontal disease in young, middle-aged, and older adults | doi:10.1902/jop.2003.74.5.610 LK - |
| Ineligible age | Aladel | 2023 | General and oral health status and malnutrition among older adults: A cross sectional study | doi:10.1016/j.clnesp.2023.09.835 LK - |
| protocol | protocol | 2017 | Protocol for a 24-Week Randomized Controlled Study of Once-Daily Oral Dose of Flax Lignan to Healthy Older Adults | doi:10.2196/resprot.6817 |
| Ineligible age | Alghofaili | 2023 | Association of Oral and General Health with Nutritional Status of Older Adults Attending Two Medical Centers in Riyadh, Saudi Arabia: A Cross-Sectional Study | doi:10.3390/nu15184032 LK - |
| Ineligible design | Alia | 2021 | The influence of age and oral health on taste perception in older adults: A case-control study | doi:10.3390/nu13114166 LK - |
| review | Alix | 2000 | Diabetes in the elderly |  |
| Ineligible age | Althaiban | 2023 | Malnutrition is associated with increased disease risk in older people in the Makkah region of Saudi Arabia: A cross-sectional study | doi:10.3389/fpubh.2023.1149739 LK - |
| Ineligible age | Aneja | 2016 | Assessment of Various Nutritional Parameters in Geriatric Patients Who underwent Different Prosthodontic Treatments |  |
| Ineligible outcome | Angst | 2024 | Oral health and nutritional status in care-dependent, community-dwelling older adults in Zurich, Switzerland | doi:10.61872/sdj-2024-02-09 LK - |
| Ineligible design | Aquilanti | 2021 | A pilot cross-sectional study on oral health and nutritional status of institutionalized older adults: A focus on sarcopenia | doi:10.3390/ijerph182413232 LK - |
| review | Walls | 1999 | Oral health and nutrition | doi:10.1093/ageing/28.5.419 |
| review | Walls and Steele | 2004 | The relationship between oral health and nutrition in older people | doi:10.1016/j.mad.2004.07.011 |
| Ineligible age | Treesattayakul | 2019 | Loss of Posterior Occluding Teeth and Its Association with Protein-Micronutrients Intake and Muscle Mass among Thai Elders: A Pilot Study | doi:10.14283/jfa.2019.2 |
| Ineligible outcome | Baggs | 2023 | Impact of a specialized oral nutritional supplement on quality of life in older adults following hospitalization: post-hoc analysis of the NOURISH trial | doi:10.1016/j.clnu.2023.09.004 |
| Ineligible age | Banerjee | 2018 | Evaluation of relationship between nutritional status and oral health related quality of life in complete denture wearers | doi:10.4103/ijdr.IJDR_285_17 LK - |
| Ineligible design | Barbe | 2018 | Xerostomia and hyposalivation in orthogeriatric patients with fall history and impact on oral health-related quality of life | doi:10.2147/CIA.S178370 LK - |
| Ineligible outcome | Barrientos-Calvo | 2012 | Prevalence of the risk of malnutrition and its main associated factors in older adults of a county of San José, Costa Rica | doi:10.1111/j.1532-5415.2012.04000.x LK - |
| review | Bell | 2023 | An Update on Drug–Nutrient Interactions and Dental Decay in Older Adults | doi:10.3390/nu15234900 LK - |
| Ineligible age | Benksim | 2021 | Self-reported morbidities, nutritional characteristics, and associated factors in institutionalized and non-institutionalized older adults | doi:10.1186/s12877-021-02067-3 LK - |
| Ineligible age | Beydoun | 2020 | Periodontal disease, sleep duration, and white blood cell markers in the 2009 to 2014 National Health and Nutrition Examination Surveys | doi:10.1002/JPER.19-0055 LK - |
| review | Bissett and Preshaw | 2011 | Guide to providing mouth care for older people | doi:10.7748/nop2011.12.23.10.14.c8837 LK - |
| cannot retrieve | Bocirnea | 2014 | Effectiveness of a nursing teaching program on oral health assessment in an acute care for elderly (ACE) unit | doi:10.1111/jgs.12870 LK - |
| Ineligible age | Borges-Yáñez | 2006 | Risk factors and prevalence of periodontitis in community-dwelling elders in Mexico | doi:10.1111/j.1600-051X.2006.00897.x LK - |
| Ineligible outcome | Borkent | 2019 | PREVALENCE AND DETERMINANTS OF UNDERNUTRITION IN A SAMPLE OF DUTCH COMMUNITY-DWELLING OLDER ADULTS: RESULTS FROM TWO ONLINE SCREENING TOOLS | doi:10.1016/S0261-5614(19)32870-5 LK - |
| Ineligible design | Borroni | 2023 | Perception Of Malnutrition In Older Adults | doi:10.1016/j.clnesp.2022.09.664 LK - |
| Ineligible age | Bradbury | 2006 | Nutrition counseling increases fruit and vegetable intake in the edentulous | doi:10.1177/154405910608500513 |
| Ineligible outcome | Braud | 2021 | Food-related oral discomfort: A cross-sectional survey assessing the sensory dimension of oral discomfort in French independently living adults | doi:10.1111/joor.13177 LK - |
| Ineligible age | Braud | 2019 | How oral health and food sensory properties impact oral comfort during consumption of apples: A comparative approach in seniors and young adults | doi:10.1111/jtxs.12450 LK - |
| review | Brígido | 2023 | The effect of prosthetic rehabilitation with or without dietary advice on nutritional status in elderly patients: a systematic review | doi:10.1007/s40520-023-02578-6 LK - |
| Ineligible design | Brodeur | 1993 | Nutrient intake and gastrointestinal disorders related to masticatory performance in the edentulous elderly |  |
| review | Budtz-Jørgensen | 2001 | Nutrition and oral health | doi:10.1053/bega.2001.0247 LK - |
| not clear age | Burks | 2017 | Risk Factors for Malnutrition among Older Adults in the Emergency Department: A Multicenter Study | doi:10.1111/jgs.14862 LK - |
| abstract only | Cuny | 2018 | Polyvalent immunoglobulins with vitamin D3 and vitamin B12 in the treatment of Sjogren's syndrome in a vegetarian with stomatitis, glossodynia, xerostomia, and elevated antinuclear antibodies: Case report | doi:10.5414/CP203143 |
| Ineligible design | Castrejón-Pérez | 2023 | Frailty index and ten oral conditions in the Coyoacan cohort study: A cross-sectional analysis | doi:10.1111/ger.12665 LK - |
| review | Besimo | 2007 | [Malnutrition in the elderly--a interdisciplinary problem for the dentist] |  |
| Ineligible outcome | Goh | 2016 | Association Between Serum Antibodies to Periodontal Bacteria and Rheumatoid Factor in the Third National Health and Nutrition Examination Survey | doi:10.1002/art.39724 |
| review | Chauhan | 2024 | Nutrition and oral health-related quality of life (OHRQoL) in older adults: a systematic review and meta-analysis | doi:10.1038/s41432-024-01088-2 LK - |
| Ineligible design | Chew | 2023 | Frailty Screening and Detection of Geriatric Syndromes in Acute Inpatient Care: Impact on Hospital Length of Stay and 30-Day Readmissions | doi:10.4235/agmr.23.0124 LK - |
| review | Cifuentes-Suazo | 2025 | Dietary Counseling: An Option to Malnutrition and Masticatory Deficiency in Patients with Total Protheses? A Scoping Review | doi:10.3390/nu17010141 LK - |
| Ineligible age | Cousson | 2012 | Nutritional status, dietary intake and oral quality of life in elderly complete denture wearers |  |
| review | Crogan | 2017 | Nutritional Problems Affecting Older Adults | doi:10.1016/j.cnur.2017.04.005 LK - |
| Ineligible age | Lin | 2023 | Association between masseter muscle volume |  |
| protocol | protocol | 2025 | Effect of Immunonutrition in perforation peritonitis patients outcome |  |
| protocol | protocol |  | Simplified complete denture quality, satisfaction, and effect on nutrition |  |
| protocol | protocol | 2017 | Importance of oral Massage and teethset on improving the eating capacity of persons with missing teeth |  |
| review | Azzolino | 2019 | Poor Oral Health as a Determinant of Malnutrition and Sarcopenia | doi:10.3390/nu11122898 |
| Ineligible design | Liu | 2024 | Self-care ability and associated factors in community-dwelling older adults living with oral frailty using the COM-B model | doi:10.1111/joor.13713 |
| Ineligible age | Rémond | 2007 | Postprandial whole-body protein metabolism after a meat meal is influenced by chewing efficiency in elderly subjects | doi:10.1093/ajcn/85.5.1286 |
| Ineligible age | Daly | 2003 | Associations between self-reported dental status and diet | doi:10.1046/j.1365-2842.2003.01169.x LK |
| Ineligible age | Damayanthi | 2018 | Prevalence of malnutrition and associated factors among community-dwelling older persons in Sri Lanka: a cross-sectional study | doi:10.1186/s12877-018-0892-2 LK - |
| Ineligible age | de Andrade | 2009 | Relationship between oral health, nutrient intake and nutritional status in a sample of Brazilian elderly people | doi:10.1111/j.1741-2358.2008.00220.x LK - |
| abstract only | de Jong | 2001 | Nutrient-dense foods and exercise in frail elderly: effects on B vitamins, homocysteine, methylmalonic acid, and neuropsychological functioning | doi:10.1093/ajcn/73.2.338 |
| ineligible age | De La Cruz Castillo-Pineda | 2020 | NUTRITIONAL STATUS AND ITS ASSOCIATION WITH ORAL HEALTH IN A SAMPLE OF OLDER ADULTS IN MORELIA, MICHOACÁN MEXICO | doi:10.1159/000511835 LK - |
| Ineligible age | De Marchi | 2012 | Number of teeth and its association with central obesity in older Southern Brazilians |  |
| Ineligible age | de Moraes Flores | 2023 | A qualitative analysis of denture wearing experience affecting the quality of life of older adults | doi:10.1007/s00784-023-04997-1 LK - |
| Ineligible design | de Oliveira | 2021 | Health-related quality of life of institutionalized older adults: Influence of physical, nutritional and self-perceived health status | doi:10.1016/j.archger.2020.104278 LK - |
| review | de Sire | 2022 | Sarcopenic Dysphagia, Malnutrition, and Oral Frailty in Elderly: A Comprehensive Review | doi:10.3390/nu14050982 LK - |
| review | De Sire | 2023 | WHICH CORRELATION AMONG SARCOPENIC DYSPHAGIA, MALNUTRITION, AND ORAL FRAILTY IN ELDERLY+ A LITERATURE OVERVIEW | doi:10.1007/s40520-023-02442-7 LK - |
| review | Desai and Nair | 2023 | Oral Health Factors Related to Rapid Oral Health Deterioration among Older Adults: A Narrative Review | doi:10.3390/jcm12093202 LK - |
| Ineligible age | Secchi | 2015 | RED MEAT, MICRONUTRIENTS AND ORAL SQUAMOUS CELL CARCINOMA OF ARGENTINE ADULT PATIENTS | doi:10.3305/nh.2015.32.3.9277 |
| Ineligible age | Dibello | 2023 | The Relationship between Oral Health-Related Quality of Life and Body Mass Index in an Older Population from Southern Italy: The Salus in Apulia Study | doi:10.3390/jpm13091300 LK - |
| protocol | protocol |  | Effects of a sugar-reduced diet on bleeding of probing after periodontal therapy |  |
| cannot retrieve | Duván Gutiérrez | 2025 | Oral health conditions and nutritional |  |
| Ineligible design | Fonad | 2015 | Associations between falls and general health, nutrition, dental health and medication use in Swedish home-dwelling people aged 75 years and over | doi:10.1111/hsc.12182 |
| Ineligible age | Eberhard | 2022 | A randomized clinical trial to investigate the effect of dietary protein sources on periodontal health | doi:10.1111/jcpe.13587 |
| ineligible age | Eberhard | 2022 | A semi-vegetarian higher fat diet improves periodontal health - a randomised clinical trial | doi:10.1111/jcpe.13636 LK - |
| not clear age | Kim | 2024 | Oral health of older Koreans by region and family status in 2020-2021 | doi:10.1111/ger.12727 |
| ineligible age | El Osta | 2013 | Relationship between oral health perception and nutritional status in senior cancer patients: Preliminary results | doi:10.1007/s00520-013-1798-3 LK - |
| Ineligible age | El Osta | 2017 | Impact of implant-supported prostheses on nutritional status and oral health perception in edentulous patients | doi:10.1016/j.clnesp.2017.01.001 LK - |
| not clear age | El Osta | 2015 | Correlation between oral health indicators and nutritional status in senior cancer patients: An observational study | doi:10.1007/s00520-015-2712-y LK - |
| Ineligible design | Elia | 2018 | Cost-effectiveness of oral nutritional supplements in older malnourished care home residents | doi:10.1016/j.clnu.2017.02.008 |
| Ineligible design | Ervin | 2008 | Healthy Eating Index scores among adults | 60 years of age and over |
| not clear age | Ervin and Dye | 2009 | The effect of functional dentition on healthy eating index scores and nutrient intakes in a nationally representative sample of older adults | doi:10.1111/j.1752-7325.2009.00124.x LK - |
| Ineligible age | Suzuki | 2021 | Relationship between Decreased Mineral Intake Due to Oral Frailty and Bone Mineral Density: Findings from Shika Study | doi:10.3390/nu13041193 |
| Ineligible age | Zhou | 2020 | The association between serum 25-hydroxyvitamin D levels and dental caries in US adults | doi:10.1111/odi.13360 |
| cannot retrieve | Fontjin-Tekamp | 1996 | The state of dentition in relation to nutrition in elderly Europeans in the SENECA Study of 1993 |  |
| Ineligible age | Fedele | 1998 | Relationship of self-reported oral health and nutritional risk among hospitalized older adults | doi:10.1097/00124784-199805000-00007 LK - |
| review | Feron | 2020 | How to propose to aging population suffering from salivation and chewing disorders innovative food products satisfying pleasure to eat and nutritional needs? Scientific strategies and approaches | doi:10.1016/j.cnd.2020.08.004 LK - |
| Ineligible age | Hakeem | 2021 | Association Between Oral Health and Frailty Among American Older Adults | doi:10.1016/j.jamda.2020.07.023 |
| Ineligible age | Fluitman | 2021 | Associations of the oral microbiota and Candida with taste, smell, appetite and undernutrition in older adults | doi:10.1038/s41598-021-02558-8 LK - |
| Ineligible age | Franco-Álvarez | 2007 | Determinants of malnutrition risk among the older adult community: A secondary analysis of the Health, Wellbeing, and Aging study (SABE) in Mexico |  |
| Ineligible age | Frydrych | 2022 | Factors influencing the cariogenic potential of oral nutritional supplements indicated for use in adults with or at risk of malnutrition | doi:10.1111/scd.12693 LK |
| review | Funke | 2023 | Impact of shortened dental arch therapy on nutritional status and treatment costs in older adults: A systematic review | doi:10.1016/j.jdent.2023.104483 LK - |
| Ineligible design | Furuta | 2018 | Tooth loss, swallowing dysfunction and mortality in Japanese older adults receiving home care services | doi:10.1111/ggi.13271 LK - |
| Ineligible age | Erdinç and Yigit | 2023 | Evaluation of the relationship between GOHAI, MNA and the dental hygiene of using denture wearers for older patients | doi:10.1111/joor.13438 |
| Ineligible age | McKenna | 2014 | Comparison of functionally orientated tooth replacement and removable partial dentures on the nutritional status of partially dentate older patients: a randomised controlled clinical trial | doi:10.1016/j.jdent.2014.03.005 |
| Ineligible design | Röhrig | 2020 | Do subjectively and objectively impaired oral health parameters influence geriatric assessment results in hospitalized geriatric patients? | doi:10.1007/s41999-020-00306-1 |
| Ineligible outcome | Ganbavale | 2025 | Association between poor oral health and deterioration of appetite in older age: Results from longitudinal analyses of two prospective cohorts from the UK and USA | doi:10.1136/bmjopen-2024-083973 LK - |
| Ineligible age | Gao | 2025 | Oral health and nutrition: addressing disparities in socioeconomically disadvantaged older adults in rural China | doi:10.1186/s12889-025-22101-y LK - |
| Ineligible age | Gonçalves | 2016 | Associations Between Caries Experience, Nutritional Status, Oral Hygiene, and Diet in a Multigenerational Cohort |  |
| Ineligible age | Gonçalves | 2015 | Effects of implant-based prostheses on mastication, nutritional intake, and oral health-related quality of life in partially edentulous patients: a paired clinical trial | doi:10.11607/jomi.3770 |
| Ineligible age | Grönstedt | 2020 | Effect of Sit-to-Stand Exercises Combined With Protein-Rich Oral Supplementation in Older Persons: the Older Person's Exercise and Nutrition Study | doi:10.1016/j.jamda.2020.03.030 |
| Ineligible age | Grootswagers | 2021 | A novel oral nutritional supplement improves gait speed and mitochondrial functioning compared to standard care in older adults with (or at risk of) undernutrition: results from a randomized controlled trial | doi:10.18632/aging.202912 |
| not clear age | Gutiérrez Quiceno | 2024 | Edentulism and Individual Factors of Active Aging Framework in Colombia | doi:10.4235/agmr.23.0158 LK - |
| review | Yamane | 2007 | [Oral care for elderly people] |  |
| Ineligible age | Flink | 2008 | Prevalence of hyposalivation in relation to general health, body mass index and remaining teeth in different age groups of adults | doi:10.1111/j.1600-0528.2008.00432.x |
| abstract only | Hägglund | 2017 | Study protocol for the SOFIA project: Swallowing function, Oral health, and Food Intake in old Age: a descriptive study with a cluster randomized trial | doi:10.1186/s12877-017-0466-8 LK - |
| Ineligible age | Hakeem | 2020 | Association between Oral Health and Frailty among Older Adults in Madinah Saudi Arabia: A Cross-Sectional Study |  |
| Ineligible age | Hanindriyo | 2018 | Influence of residential characteristics on the association between the oral health status and BMI of older adults in Indonesia | doi:10.1111/ger.12352 LK - |
| not clear age | Hanioka | 2007 | Association of total tooth loss with smoking, drinking alcohol and nutrition in elderly Japanese: analysis of national database | doi:10.1111/j.1741-2358.2007.00166.x LK - |
| Ineligible age | Hasegawa and Watase | 2004 | Multiple risk factors of periodontal disease: A study of 9260 Japanese non-smokers | doi:10.1111/j.1447-0594.2003.00116.x LK - |
| Ineligible age | Higashi | 2023 | The relationship between changes in occlusal support and masticatory performance using 6-year longitudinal data from the SONIC study | doi:10.1016/j.jdent.2023.104763 LK - |
| Ineligible age | Hilgert | 2009 | Oral status and its association with obesity in Southern Brazilian older people | doi:10.1111/j.1741-2358.2008.00226.x LK - |
| not clear age | Lee | 2010 | More missing teeth are associated with poorer general health in the rural Korean elderly | doi:10.1016/j.archger.2009.01.005 |
| Ineligible age | Chen | 2024 | Association of dietary inflammation with tooth loss and cognitive decline in older adults from cross-sectional data: The moderated role of albumin | doi:10.1016/j.jdent.2024.104967 |
| not clear age | Hollaar | 2024 | Associations Between Problems in Oral Health, Oral Function and Malnutrition in Older People: Results From Three Databases | doi:10.1111/idh.12886 LK - |
| Ineligible design | Hollaar | 2024 | Success factors and barriers in interprofessional collaboration between dental hygienists and dietitians in community-dwelling older people: Focus group interviews | doi:10.1111/idh.12774 LK - |
| Ineligible age | Honeywell | 2022 | Associations between Dentition Status and Nutritional Status in Community-Dwelling Older Adults | doi:10.1177/23800844211063859 LK - |
| Ineligible age | Hung | 2019 | Appetite and Protein Intake Strata of Older Adults in the European Union: Socio-Demographic and Health Characteristics, Diet-Related and Physical Activity Behaviours | doi:10.3390/nu11040777 LK - |
| Ineligible age | Huraib | 2022 | Assessment of nutritional and psychosocial status of elderly patients wearing removable dental prosthetics | doi:10.4103/jpbs.jpbs-840-21 LK - |
| review | Hussein | 2022 | Association of Oral Health and Mini Nutritional Assessment in Older Adults: A Systematic Review with Meta-analyses | doi:10.2186/jpr.JPR_D_20_00207 LK - |
| protocol | Nykänen | 2021 | Optimizing nutrition and oral health for caregivers-intervention protocol | doi:10.1186/s13063-021-05589-8 |
| review | Prêcheur and Chevalier | 2015 | [The fight against malnutrition in older adults: new aproaches in dentistry] | doi:10.1684/pnv.2015.0515 |
| Ineligible outcome | Ikebe | 2014 | The relationship between oral function and body mass index among independently living older Japanese people |  |
| Ineligible outcome | Inamoto | 2025 | Oral Health Management by Dental Hygienists and Older Adults' Food Forms in Long-Term Care Facilities: A 1-Year Longitudinal Study | doi:10.1111/ger.12810 LK - |
| Ineligible outcome | Inomata | 2017 | Dietary Intake Is Associated with Occlusal Force Rather Than Number of Teeth in 80-y-Old Japanese | doi:10.1177/2380084416673963 LK - |
| Ineligible age | Ishimiya | 2018 | Tooth loss-related dietary patterns and cognitive impairment in an elderly Japanese population: The nakajima study | doi:10.1371/journal.pone.0194504 LK - |
| Ineligible design | Ito | 2019 | Qualitative analysis of the vocabulary used in work logs of a preventive programme for elderly oral function and nutrition | doi:10.1111/joor.12804 |
| Ineligible design | Iwasaki | 2011 | Relationship between saturated fatty acids and periodontal disease | doi:10.1177/0022034511405384 LK - |
| Ineligible age | Iwasaki | 2022 | Oral hypofunction and malnutrition among community-dwelling older adults: Evidence from the Otassha study | doi:10.1111/ger.12580 LK - |
| review | Aida | 2022 | Oral Health and Nutrition: Epidemiology, Clinical, and Social Aspects | doi:10.3177/jnsv.68.S26 |
| Ineligible age | Botelho | 2021 | The Role of Inflammatory Diet and Vitamin D on the Link between Periodontitis and Cognitive Function: A Mediation Analysis in Older Adults | doi:10.3390/nu13030924 |
| cannot retrieve | Chai | 2006 | Influence of dental status on nutritional status of geriatric patients in a convalescent and rehabilitation hospital |  |
| review | Mills and Schuman | 1999 | A clinical approach to dental nutrition among the elderly: a description and discussion of geriatric dental nutrition |  |
| review | Prpić | 2012 | Obesity and oral health--is there an association? |  |
| Ineligible age | Brígido | 2025 | Influence of prosthodontic rehabilitation and dietary advice on glycaemic control nutritional status and oral health-related quality of life of older adults with type 2 diabetes |  |
| Ineligible age | Gil-Montoya | 2008 | Oral health-related quality of life and nutritional status | doi:10.1111/j.1752-7325.2007.00082.x |
| Ineligible age | Canaan | 2023 | Food preferences and periodontal status of adults assisted by a public health care system | doi:10.1371/journal.pone.0291878 |
| Ineligible age | Steele | 2001 | Clinical and behavioural risk indicators for root caries in older people | doi:10.1111/j.1741-2358.2001.00095.x |
| Ineligible age | Lee | 2023 | The association of food insecurity with tooth decay and periodontitis among middle-aged and older adults in the United States and Korea | doi:10.1111/ger.12651 |
| Ineligible design | Chalmers | 2002 | Caries experience in existing and new nursing home residents in Adelaide, Australia | doi:10.1111/j.1741-2358.2002.00030.x |
| Ineligible outcome | Starr | 2008 | Predictors and correlates of edentulism in the healthy old people in Edinburgh (HOPE) study | doi:10.1111/j.1741-2358.2008.00227.x |
| Ineligible outcome | Joosten and Vander Elst | 2001 | Does nutritional supplementation influence the voluntary dietary intake in an acute geriatric hospitalized population? | doi:10.1007/bf03351508 |
| review | O'Conner | 2020 | Poor dietary intake of nutrients and food groups are associated with increased risk of periodontal disease among community-dwelling older adults: a systematic literature review | doi:10.1093/nutrit/nuz035 |
| Ineligible outcome | Jung | 2024 | Smartphone-based combined oral and whole-body exercise programme aimed at improving oral functions: a randomized clinical trial | doi:10.1111/idh.12807 |
| Ineligible age | Albright | 2013 | Synergism between obesity and poor oral health associated with diabetes in an elderly human population |  |
| Ineligible outcome | Ikebe | 2018 | Occlusal force is correlated with cognitive function directly as well as indirectly via food intake in community-dwelling older Japanese: From the SONIC study | doi:10.1371/journal.pone.0190741 |
| Ineligible age | Kilic | 2021 | Influence of Conventional Complete Dentures and Different Attachment Types in Implant-Supported Overdentures on Quality of Life and Nutritional Status in Edentulous Geriatric Patients | doi:10.11607/ijp.6690 |
| Ineligible outcome | Suzuki | 2005 | Relationship between number of present teeth and nutritional intake in institutionalized elderly | doi:10.2209/tdcpublication.46.135 |
| Ineligible design | Ueda | 2003 | A study on the effects of short-, medium- and long-term professional oral care in elderly persons requiring long-term nursing care at a chronic or maintenance stage of illness | doi:10.1111/j.1741-2358.2003.00050.x |
| Ineligible design | Kaipainen | 2024 | Effect of individually tailored nutritional counseling on frailty status in older adults with protein-energy malnutrition or risk of it: an intervention study among home care clients | doi:10.1038/s41430-024-01547-0 LK - |
| Ineligible outcome | Kaipainen | 2018 | Individually tailored dietary counseling among old home care clients: Effects on nutrient intake | doi:10.1007/s41999-018-0097-4 LK - |
| Ineligible age | Kalavathy | 2022 | Effect of serum vitamin D calcium |  |
| Ineligible outcome | Karawekpanyawong | 2023 | Oral Health and Nutritional Intake in Community-Dwelling 90-Year-Old Japanese People: A Cross-Sectional Study | doi:10.1111/ger.12627 LK - |
| Protocol | Keller | 2017 | Making the Most of Mealtimes (M3): protocol of a multi-centre cross-sectional study of food intake and its determinants in older adults living in long term care homes | doi:10.1186/s12877-016-0401-4 LK - |
| review | Kelsall and O'Keefe | 2014 | Good health requires a healthy mouth: Improving the oral health of Canada's seniors | doi:10.1503/cmaj.140975 LK - |
| Ineligible age | Khan | 2023 | Assessment of nutritional status using anthropometric index among older adult and elderly population in India | doi:10.1038/s41598-023-39167-6 LK - |
| Ineligible age | Kiesswetter | 2019 | Oral health determinants of incident malnutrition in community-dwelling older adults | doi:10.1016/j.jdent.2019.05.017 LK - |
| ineligible age | Kiesswetter | 2018 | The association of oral health with body weight within 10 years in community-dwelling older adults | doi:10.1007/s41999-018-0097-4 LK - |
| Ineligible age | Kilpinen-Loisa | 2009 | Vitamin D status and optimal supplementation in institutionalized adults with intellectual disability | doi:10.1111/j.1365-2788.2009.01218.x |
| review | Kiss | 2016 | Nutrition and Oral Health in the Elderly | doi:10.1055/s-0041-111331 LK - |
| Ineligible age | Kiuchi | 2022 | Oral Status and Dementia Onset: Mediation of Nutritional and Social Factors | doi:10.1177/00220345211049399 LK - |
| Ineligible design | Koponen | 2023 | Depressive symptoms, low-grade inflammation, and poor oral health prevents the success of nutritional guidance in older caregivers | doi:10.1016/j.clnesp.2023.06.018 LK - |
| review | Kossioni | 2018 | The association of poor oral health parameters with malnutrition in older adults: A review considering the potential implications for cognitive impairment | doi:10.3390/nu10111709 LK - |
| Ineligible age | Kshetrimayum | 2013 | Oral health-related quality of life and nutritional status of institutionalized elderly population aged 60 years and above in Mysore City, India | doi:10.1111/j.1741-2358.2012.00651.x LK - |
| Ineligible age | Küçük | 2024 | Nutrition, Chewing Ability and Quality of Life in Older Adults With Implant Overdentures: A Prospective Clinical Study | doi:10.1111/ger.12802 LK - |
| Ineligible outcome | Kusama | 2023 | Dental prosthesis use is associated with higher protein intake among older adults with tooth loss | doi:10.1111/joor.13554 LK - |
| Ineligible age | Kusama | 2023 | Weight Loss Mediated the Relationship between Tooth Loss and Mortality Risk | doi:10.1177/00220345221120642 LK - |
| Ineligible age | Kusama | 2024 | The association between objective and subjective oral health conditions and the presence of anorexia of aging among Japanese older Adults1 | doi:10.1016/j.appet.2024.107332 LK - |
| Ineligible outcome | Kwok | 2004 | Association between functional dental state and dietary intake of Chinese vegetarian old age home residents | doi:10.1111/j.1741-2358.2004.00030.x LK - |
| Ineligible age | Kwong | 2024 | Race and gender disparity of dietary lycopene intake and periodontitis severity in older adults | doi:10.1101/2024.05.29.24308111 LK - |
| Ineligible age | Jamieson | 2024 | Oral health inequities over time among older adults of different racial/ethnic backgrounds: a comparative decomposition analysis across Australia and the United States | doi:10.1186/s12877-024-05525-w |
| Ineligible age | Jauhiainen | 2017 | Food Consumption and Nutrient Intake in Relation to Denture Use in 55- to 84-Year-Old Men and Women -Results of a Population Based Survey | doi:10.1007/s12603-016-0793-z |
| Ineligible age | Sahab | 2025 | The Nutritional Pathway Between Tooth Loss and Healthy Ageing: A Longitudinal Study of Older American Adults | doi:10.3390/nu17040719 |
| Ineligible age | Lahoud | 2024 | Association between weight-adjusted waist index and periodontitis: A population-based study | doi:10.1371/journal.pone.0305010 |
| review | Lahoud | 2023 | Masticatory dysfunction in older adults: A scoping review | doi:10.1111/joor.13493 LK - |
| Ineligible age | Lee | 2022 | Gender differences in the association between oral health literacy and oral health-related quality of life in older adults | doi:10.1186/s12903-022-02237-8 LK - |
| Ineligible age | Lee | 2004 | Edentulism and nutritional status in a biracial sample of well-functioning, community-dwelling elderly: The Health, Aging, and Body Composition Study | doi:10.1093/ajcn/79.2.295 LK - |
| Ineligible age | LH | 2013 | Association between underweight and overweight/obesity with oral health among independently living Brazilian elderly | doi:10.1016/j.nut.2012.05.011 |
| Ineligible age | Li | 2023 | Association of Serum Vitamin D With Periodontal Disease | doi:10.1016/j.identj.2023.06.004 LK - |
| Ineligible design | Lin | 2021 | Effects of a food preparation program on dietary well-being for stroke patients with dysphagia: a pilot study | doi:10.1097/MD.0000000000026479 |
| Ineligible outcome | Liu | 2018 | THE ASSOCIATION BETWEEN EATING PERFORMANCE AND DENTAL-RELATED FUNCTION AND ORAL HEALTH IN COGNITIVELY IMPAIRED RESIDENTS IN ASSISTED LIVING SETTINGS | doi:10.1016/j.jalz.2018.06.1879 LK - |
| Ineligible age | Logan | 2018 | A longitudinal investigation of whether oral health status has an impact on future dietary intake in older adults in Northern Ireland | doi:10.1017/S002966511800112X LK - |
| Ineligible age | LP | 1995 | The dietary adequacy of edentulous older adults | doi:10.1016/s0022-3913(05)80153-2 |
| Ineligible age | Lu and Gao | 2024 | Association Between `Life's Essential 8' and Periodontitis: A Cross-Sectional Study From the NHANES Database | doi:10.1016/j.identj.2024.03.011 LK - |
| Ineligible age | Adiatman | 2013 | Functional tooth units and nutritional status of older people in care homes in Indonesia | doi:10.1111/j.1741-2358.2012.00673.x |
| Ineligible outcome | Aishima | 2023 | Unplanned Hospital Visits and Poor Oral Health With Undernutrition in Nursing Home Residents | doi:10.1016/j.jamda.2023.07.013 |
| Ineligible age | Hung | 2024 | The Association of Vitamin D Levels and Dental Caries in Older Adults: A Cross-Sectional Study | doi:10.3390/nu16142307 |
| Ineligible age | Iwasaki | 2010 | Longitudinal relationship between dietary _-3 fatty acids and periodontal disease | doi:10.1016/j.nut.2009.09.010 |
| Ineligible age | Iwasaki | 2023 | Serum levels of vitamin D and periodontal inflammation in community-dwelling older Japanese adults: The Otassha Study | doi:10.1111/jcpe.13834 |
| Ineligible age | Jimenez | 2014 | Predicted vitamin D status and incidence of tooth loss and periodontitis | doi:10.1017/S1368980013000177 |
| review | Liu | 2024 | [Research Progress on the Correlation Between Oral Frailty and Dietary Intake in Older Adults] | doi:10.12182/20240960106 |
| Ineligible outcome | Liu | 2024 | A study on the reasonable dietary trajectory of elderly people in the community and its correlation with body mass index | doi:10.3389/fnut.2024.1429627 |
| review | O'Keeffe | 2019 | Potentially modifiable determinants of malnutrition in older adults: A systematic review | doi:10.1016/j.clnu.2018.12.007 |
| Ineligible design | Yücel | 2025 | A comparison of oral health, nutrition, and swallowing function in older adults with and without sarcopenia: A cross‐sectional study | doi:10.1002/ncp.11283 |
| Ineligible age | Mack | 2008 | The factors associated with body mass index in adults from the study of health in Pomerania (SHIP-0), Germany |  |
| not clear age | Madarász | 2019 | PAM14 ASSESSING THE NUTRITIONAL STATUS OF THE ELDERLY USING STANDARD STATUS SCALES | doi:10.1016/j.jval.2019.09.102 LK - |
| not clear age | Makhija | 2007 | Association between aspects of oral health-related quality of life and body mass index in community-dwelling older adults | doi:10.1111/j.1532-5415.2007.01391.x LK - |
| not clear age | Mameno | 2024 | Exploring the association between number of teeth, food intake, and cognitive function: A 9-year longitudinal study | doi:10.1016/j.jdent.2024.104991 LK - |
| Ineligible age | Marcenes | 2003 | The relationship between dental status, food selection, nutrient intake, nutritional status, and body mass index in older people. |  |
| Ineligible age | Marito | 2022 | The Association of Dietary Intake Oral Health |  |
| Ineligible design | Matsuda | 2022 | Effect of the COVID-19 pandemic on the oral health and nutritional status of Japanese older adults who underwent total hip or knee arthroplasty: A 3-year single-institution retrospective cohort study | doi:10.1097/MD.0000000000031937 LK - |
| abstract only | Matsuo | 2019 | Impact of deterioration of oral health and eating function on the general health and QOL |  |
| Ineligible age | McCrum | 2019 | Dietary intervention coupled with oral rehabilitation in partially dentate elders |  |
| duplication | McCrum | 2020 | A tailored habits-based dietary intervention combined with oral rehabilitation on partially dentate older adults: Nutritional impact | doi:10.1017/S0029665120000968 LK - |
| Ineligible age | McCrum | 2020 | Development and feasibility of a tailored habit-based dietary intervention coupled with natural tooth replacement on the nutritional status of older patients | doi:10.1186/s40814-020-00654-6 LK - |
| review | McGowan | 2019 | Impact of behavioural dietary intervention coupled with oral rehabilitation on the health and nutritional status of older adults: A systematic review and meta-analysis | doi:10.1159/000489691 LK - |
| review | McGowan | 2020 | The impact of oral rehabilitation coupled with healthy dietary advice on the nutritional status of adults: A systematic review and meta-analysis | doi:10.1080/10408398.2019.1630600 LK |
| Ineligible age | McKay | 2012 | Effects of whole grain phytochemicals on biomarkers of postprandial metabolic dysregulation in overweight/obese adults following an oral glucose challenge |  |
| not clear age | McKenna | 2012 | The effect of tooth replacement strategies on the nutritional status of older patients | doi:10.1007/s11845-012-0842-5 LK - |
| Ineligible age | McKenna | 2012 | Impact of tooth replacement strategies on the nutritional status of partially-dentate elders |  |
| Ineligible outcome | McNeill | 2007 | Effect of multivitamin and multimineral supplementation on cognitive function in men and women aged 65 years and over: a randomised controlled trial | doi:10.1186/1475-2891-6-10 |
| Ineligible age | Irigoyen-Camacho | 2024 | Physical Performance, Body Composition, and Oral Health in Community-Residing Older Adults: A Cross-Sectional Study | doi:10.3390/geriatrics9040089 |
| Ineligible age | Meisel | 2021 | Tooth loss and adiposity: possible role of carnitine transporter (OCTN1/2) polymorphisms in women but not in men | doi:10.1007/s00784-020-03594-w LK - |
| Ineligible age | Kang and Jung | 2022 | Association Between Oral Health and Frailty in Older Korean Population: A Cross-Sectional Study | doi:10.2147/CIA.S384417 |
| Ineligible design | Midttun | 2024 | Beneficial effects of exercise, testosterone, vitamin D, calcium and protein in older men—A randomized clinical trial | doi:10.1002/jcsm.13498 |
| Ineligible age | Mitri | 2017 | Determinants of the nutritional status of older adults in urban Lebanon | doi:10.1111/ggi.12732 LK - |
| Ineligible design | Saunders | 1995 | Incorporating the nutrition screening initiative into the dental practice | doi:10.1111/j.1754-4505.1995.tb00468.x |
| Ineligible age | Morais | 2003 | The effects of mandibular two-implant overdentures on nutrition in elderly edentulous individuals | doi:10.1177/154405910308200112 |
| cannot retrieve | Moynihan | 2023 | Eating-related quality of life in people who wear complete dentures | doi:10.1017/S0029665123000848 LK - |
| review | Moynihan | 2007 | The relationship between nutrition and systemic and oral well-being in older people | doi:10.14219/jada.archive.2007.0201 LK - |
| review | Moynihan and Teo | 2024 | Exploring Oral Function, Protein Intake, and Risk of Sarcopenia: A Scoping Review | doi:10.1177/23800844231157259 LK - |
| review | Toniazzo | 2018 | Relationship of nutritional status and oral health in elderly: Systematic review with meta-analysis | doi:10.1016/j.clnu.2017.03.014 |
| not clear age | Lee | 2021 | Relationship between Vitamin D Deficiency and Periodontitis in Korean Adults Aged ≥60 Years: Analysis of Data from the Korea National Health and Nutrition Examination Survey (2013-2014) | doi:10.3390/ijerph18084181 |
| Ineligible age | Savoca | 2010 | Association between dietary quality of rural older adults and self-reported food avoidance and food modification due to oral health problems | doi:10.1111/j.1532-5415.2010.02909.x |
| Ineligible age | Savoca | 2011 | Impact of denture usage patterns on dietary quality and food avoidance among older adults | doi:10.1080/01639366.2011.545043 |
| review | Müller and Nitschke | 2005 | Oral health | dental state and nutrition in older adults |
| review | Müller and Schimmel | 2010 | Tooth loss and dental prostheses in the oldest old | doi:10.1016/j.eurger.2010.06.001 LK - |
| review | Müller | 2017 | Oral health for an ageing population: the importance of a natural dentition in older adults | doi:10.1111/idj.12329 LK - |
| Ineligible age | Khongsirisombat | 2022 | Increased Oral Dryness and Negative Oral Health-Related Quality of Life in Older People with Overweight or Obesity | doi:10.3390/dj10120231 |
| Ineligible design | Shah | 2004 | Edentulousness denture wear and denture needs of Indian elderly--a community-based study |  |
| Ineligible outcome | Naito | 2010 | Effects of dental treatment on the quality of life and activities of daily living in institutionalized elderly in Japan | doi:10.1016/j.archger.2009.01.013 |
| Ineligible age | Nakamura | 2024 | Public Health | doi:10.1002/alz.088654 |
| not clear age | Nakamura | 2016 | Poor Oral Health and Diet in Relation to Weight Loss, Stable Underweight, and Obesity in Community-Dwelling Older Adults: A Cross-Sectional Study From the JAGES 2010 Project | doi:10.2188/jea.JE20150144 LK - |
| Ineligible design | Nanayakkara | 2019 | From menu to mouth: The decay pathway of nutrient intake from planned menu to consumed and characteristics of residents in an aged care facility with greater nutrient decay rates: A cross-sectional study | doi:10.1136/bmjopen-2018-024044 LK - |
| abstract only | Nandhini | 2025 | Do implant overdentures improve chewing ability and quality of life despite no effect on nutritional status? | doi:10.1038/s41432-025-01107-w LK - |
| Ineligible design | Nanri | 2019 | Consumption of green tea but not coffee is associated with the oral health-related quality of life among an older Japanese population: Kyoto-Kameoka cross-sectional study | doi:10.1038/s41430-018-0186-y LK - |
| Ineligible age | Nascimento | 2022 | Relationship between tooth loss, nutritional status and consumption of macro and micronutrients in elderly people from southern Brazil | doi:10.4025/actascihealthsci.v44i1.53676 LK - |
| Ineligible design | Naumann | 2024 | Collaboration between Dental Hygienists and Dietitians in the Care for Community Dwelling Older Adults | doi:10.1159/000535909 LK - |
| Ineligible age | Nazliel | 2012 | Oral health status in a group of the elderly population residing at home |  |
| review | Nazri | 2021 | Malnutrition, low diet quality and its risk factors among older adults with low socio-economic status: a scoping review | doi:10.1017/S0954422420000189 LK - |
| protocol | NCT02315222 |  | Dietary Supplements and Periodontal Wound Healing |  |
| protocol | NCT03613116 |  | High-dose Vitamin D Supplements in Older Adults |  |
| protocol | NCT04734951 | 2020 | Muscle Quality Index Improvement by Exercise and HMB Oral Supplementation in Older Adults |  |
| protocol | NCT05482373 | 2022 | Adjuvant Treatment in Elderly Patients With Periodontitis Through the Administration of a Supplement Rich in Oleuropein From the Olive Leaf |  |
| protocol | NCT05724043 | 2023 | SmartJournal for Improved Oral Care in Nursing Homes |  |
| Ineligible age | Neelemaat | 2012 | Oral nutritional support in malnourished elderly decreases functional limitations with no extra costs | doi:10.1016/j.clnu.2011.10.009 |
| Ineligible design | Neziraj | 2023 | The STAIR OF KNOWLEDGE-a codesigned intervention to prevent pressure ulcers | malnutrition |
| Ineligible design | Neziraj | 2021 | Prevalence of risk for pressure ulcers | malnutrition |
| duplication | Nihtilä | 2017 | The effect of individually tailored dietary and oral health counselling among older home care clients on xerostomia |  |
| review | Nitsuwat | 2025 | The Association of Oral Processing Factors and Nutrient Intake in Community-Dwelling Older Adults: A Systematic Review and Meta-Analysis | doi:10.1093/nutrit/nuae080 LK |
| Ineligible age | Rhodus | 1990 | Nutritional intake in both free-living and institutionalized older adults with xerostomia | doi:10.1300/j052v10n01_01 |
| cannot retrieve | Rhodus and Brown | 1990 | The association of xerostomia and inadequate intake in older adults |  |
| Ineligible outcome | Nóbrega | 2024 | Tolerability of glutamine supplementation in older adults: a double-blind placebo-controlled randomized clinical trial | doi:10.1590/1414-431X2024e13468 |
| Ineligible design | Nomura | 2020 | Impact of the serum level of albumin and self-assessed chewing ability on mortality |  |
| Ineligible age | Noriko | 2022 | Oral Factors as Predictors of Frailty in Community-Dwelling Older People: A Prospective Cohort Study | doi:10.3390/ijerph19031145 LK - |
| Ineligible age | Sahyoun | 2003 | Nutritional status of the older adult is associated with dentition status | doi:10.1053/jada.2003.50003 |
| Ineligible age | Sahyoun and krall | 2003 | Low dietary quality among older adults with self-perceived ill-fitting dentures | doi:10.1016/j.jada.2003.08.025 |
| Ineligible age | Sahyoun | 2005 | Barriers to the consumption of fruits and vegetables among older adults | doi:10.1300/j052v24n04_03 |
| Ineligible design | Ogawa | 2006 | Association between serum albumin and periodontal disease in community-dwelling elderly | doi:10.1111/j.1600-051X.2005.00901.x LK - |
| Ineligible design | Oikarinen | 2014 | Body mass index and periodontal infection in a sample of non-smoking older individuals | doi:10.1111/odi.12108 LK - |
| not clear age | Okabe | 2016 | Swallowing function and nutritional status in Japanese elderly people receiving home-care services: A 1-year longitudinal study | doi:10.1007/s12603-015-0645-2 LK - |
| duplication | Oliveira | 2020 | Comparison of Portuguese and Turkish older adults' nutritional status assessed by MNA-FF | doi:10.1017/S0029665120005686 LK - |
| Ineligible design | Oliveira | 2020 | Edentulism and all-cause mortality among Brazilian older adults: 11-years follow-up | doi:10.1590/1807-3107bor-2020.vol34.0046 LK - |
| abstract only | Burress | 2015 | Relationship between dental health and nutrition risk factors in homebound older adults | doi:10.1111/jgs.13275 |
| Ineligible age | Öztürk | 2023 | Nutritional Status among Portuguese and Turkish Older Adults Living in the Community: Relationships with Sociodemographic | Health and Anthropometric Characteristics |
| Ineligible age | Gaewkhiew | 2019 | Functional dentition | dietary intake and nutritional status in Thai older adults |
| Ineligible outcome | Ke | 2025 | Does low body weight mediate the relationship between tooth loss and cognitive impairment? A longitudinal cohort study of an older Chinese population | doi:10.1186/s12877-025-05750-x |
| Ineligible age | Srisilapanan | 2002 | Number of teeth and nutritional status in Thai older people |  |
| Ineligible age | Pancheva | 2018 | Nutrition in subjects with complete dentures: Energy and macronutrient intake | doi:10.5272/jimab.2018243.2104 LK - |
| Ineligible age | Peng | 2021 | Oral Nutritional Supplement with β-hydroxy-β-methylbutyrate (HMB) Improves Nutrition, Physical Performance and Ameliorates Intramuscular Adiposity in Pre-Frail Older Adults: a Randomized Controlled Trial | doi:10.1007/s12603-021-1621-7 |
| Ineligible age | Perera and Ekanayake | 2012 | Relationship between nutritional status and tooth loss in an older population from Sri Lanka |  |
| ineligible age | Popova | 2022 | Correlation of dietary intake of vitamin D and dental caries in adults living with obesity | doi:10.5272/jimab.2022Supplement2 LK - |
| cannot retrieve | Poulsen | 2006 | Nutritional status and associated factors on geriatric admission |  |
| not clear age | Prakash | 2012 | Nutritional status assessment in complete denture wearers |  |
| Ineligible age | Quandt and Chao | 2000 | Gender differences in nutritional risk among older rural adults | doi:10.1177/073346480001900202 LK - |
| Ineligible outcome | Suzuki | 2015 | Prognosis-related factors concerning oral and general conditions for homebound older adults in Japan | doi:10.1111/ggi.12382 |
| Ineligible age | Rahman | 2021 | Factors associated with the nutritional status of the older population in a selected area of Dhaka, Bangladesh | doi:10.1186/s12877-021-02068-2 LK - |
| Ineligible age | Rajasekar | 2025 | Correlation of Body Mass Index With Severity of Periodontitis: A Cross-Sectional Study | doi:10.1002/cre2.70058 LK - |
| Ineligible design | Ramage-Morin | 2010 | Health-promoting factors and good health among Canadians in mid- to late life |  |
| not clear age | Ervin and Dye | 2012 | Number of natural and prosthetic teeth impact nutrient intakes of older adults in the United States | doi:10.1111/j.1741-2358.2011.00546.x |
| protocol | protocol | 2020 | Evaluation of two different approaches in mandibular edentulous type 2 diabetic patients rehabilitated through overdentures: 2-years outcomes of a split-mouth RCT |  |
| protocol | protocol | 2013 | Rehabilitation of the Mastication in Patients with Different Types of Dental Prostheses |  |
| protocol | protocol | 2020 | Effects of Nutritional Supplementation on older seniors |  |
| Ineligible age | de Araújo | 2024 | Relationship between oral status | swallowing function |
| Ineligible design | Reinders | 2019 | Effectiveness of nutritional interventions in older adults at risk of malnutrition across different health care settings: pooled analyses of individual participant data from nine randomized controlled trials | doi:10.1016/j.clnu.2018.07.023 |
| Ineligible design | Renvert | 2013 | Tooth loss and periodontitis in older individuals: Results from the Swedish National Study on aging and care | doi:10.1902/jop.2012.120378 LK - |
| duplication | Richmond | 2016 | Modifiable risk factors for malnutrition among older adults receiving care in the emergency department | doi:10.1111/acem.12974 LK - |
| Ineligible design | Ritter-Gooder | 2011 | Content Validation of a Standardized Language Diagnosis by Certified Specialists in Gerontological Nutrition | doi:10.1016/j.jada.2011.01.012 LK - |
| Ineligible age | Marchi | 2008 | Association between oral health status and nutritional status in south Brazilian independent-living older people | doi:10.1016/j.nut.2008.01.054 |
| Ineligible age | Marchi | 2012 | Association between number of teeth, edentulism and use of dentures with percentage body fat in south Brazilian community-dwelling older people | doi:10.1111/j.1741-2358.2010.00411.x |
| Ineligible age | RL | 2004 | Persistent oral health problems associated with comorbidity and impaired diet quality in older adults | doi:10.1016/j.jada.2004.05.210 |
| Ineligible outcome | Rondanelli | 2011 | Effect of essential amino acid supplementation on quality of life | amino acid profile and strength in institutionalized elderly patients |
| ineligible age | Rosli | 2017 | H6: Association between oral health conditions, oral health-related quality of life and nutritional status among older adults |  |
| Ineligible age | Techapiroontong | 2022 | The impact of poor dental status and removable dental prosthesis quality on body composition, masticatory performance and oral health-related quality of life: a cross-sectional study in older adults | doi:10.1186/s12903-022-02103-7 |
| Ineligible age | Vijaya | 2024 | Effectiveness of dietary intervention in geriatric patients receiving new complete dentures: A randomized controlled trial | doi:10.4103/jips.jips_166_24 |
| Ineligible age | Wagley | 2015 | PERIODONTAL DISEASE AND AGE-RELATED MACULAR DEGENERATION: Results From the National Health and Nutrition Examination Survey III | doi:10.1097/IAE.0000000000000427 |
| Ineligible age | Borges-Yañez | 2004 | Dietary fiber intake and dental health status in urban-marginal | and rural communities in central Mexico |
| Ineligible age | Quandt | 2009 | Disparities in oral health status between older adults in a multiethnic rural community: the rural nutrition and oral health study | doi:10.1111/j.1532-5415.2009.02367.x |
| Ineligible age | Quandt | 2010 | Food avoidance and food modification practices of older rural adults: association with oral health status and implications for service provision | doi:10.1093/geront/gnp096 |
| Ineligible age | Quandt | 2011 | Dry mouth and dietary quality in older adults in north Carolina | doi:10.1111/j.1532-5415.2010.03309.x |
| Ineligible design | Sadarangani | 2019 | Racial Disparities in Nutritional Risk among Community-Dwelling Older Adults in Adult Day Health Care | doi:10.1080/21551197.2019.1647327 LK - |
| Ineligible design | Saito | 2019 | Number of Teeth, Oral Self-care, Eating Speed, and Metabolic Syndrome in an Aged Japanese Population | doi:10.2188/jea.JE20170210 LK - |
| Ineligible age | Saksono | 2019 | Relationships between tooth loss and masticatory performance, nutrition intake, and nutritional status in the elderly | doi:10.4034/PBOCI.2019.191.148 LK - |
| Ineligible age | Samnieng | 2012 | Relationships Between Tooth Loss and Masticatory Performance, Nutrition Intake, and Nutritional Status in the Elderly |  |
| Ineligible age | Samnieng | 2011 | Oral health status and chewing ability is related to mini-nutritional assessment results in an older adult population in Thailand | doi:10.1080/21551197.2011.591271 LK - |
| Ineligible age | Santos | 2024 | Socio-economic inequalities in oral health among Portuguese older adults: a cross-sectional study | doi:10.1186/s12889-024-21049-9 LK - |
| not clear age | Savoca | 2010 | Severe tooth loss in older adults as a key indicator of compromised dietary quality | doi:10.1017/S1368980009991236 LK - |
| cannot retrieve | Sawa | 2014 | Food-texture modification is associated with nutritional status, activities of daily living, and swallowing ability: Cross-sectional study of Japanese community-dwelling older adults |  |
| Ineligible age | Sawada | 2022 | Oral function, nutritional status and physical status in Japanese independent older adults | doi:10.1111/ger.12593 LK - |
| Ineligible age | Sawada | 2024 | Effect of oral health status and oral function on malnutrition in community-dwelling older adult dental patients: A two-year prospective cohort study | doi:10.1111/ger.12718 LK - |
| Ineligible design | Shah | 2003 | Gender issues and oral health in elderly Indians | doi:10.1002/j.1875-595X.2003.tb00890.x LK - |
| Ineligible design | Shah and Sundaram | 2003 | Impact of socio-demographic variables, oral hygiene practices and oral habits on periodontal health status of Indian elderly: a community-based study |  |
| not clear age | Sheiham and Steele | 2001 | Does the condition of the mouth and teeth affect the ability to eat certain foods, nutrient and dietary intake and nutritional status amongst older people? | doi:10.1079/phn2000116 LK - |
| not clear age | Sheiham | 2002 | The relationship between oral health status and Body Mass Index among older people: a national survey of older people in Great Britain |  |
| not clear age | Sheiham | 2001 | The Relationship among Dental Status, Nutrient Intake, and Nutritional Status in Older People | doi:10.1177/00220345010800020201 LK - |
| not clear age | Sheng | 2018 | Correlation between oral health and quality of life among the elderly in Southwest China from 2013 to 2015 | doi:10.1097/MD.0000000000010777 LK - |
| review | Shepherd | 2002 | The impact of oral health on nutritional status |  |
| ineligible age | Shigli and Hebbal | 2012 | Does prosthodontic rehabilitation change the eating patterns among completely edentulous patients? | doi:10.1111/j.1741-2358.2010.00404.x LK - |
| review | Shiohama and Wakabayashi | 2018 | No clinically significant effect of new complete denture fabrication and simple dietary advice on nutrient intake and masticatory function of edentulous older people | doi:10.1016/j.clnu.2017.11.015 LK - |
| ineligible age | Shiota | 2023 | Oral Hypofunction and Risk of Weight Change among Independent Older Adults | doi:10.3390/nu15204370 LK - |
| review | Ship | 1996 | Geriatric oral health and its impact on eating | doi:10.1111/j.1532-5415.1996.tb06419.x LK - |
| review | Shiraishi | 2020 | Oral Management in Rehabilitation Medicine: Oral Frailty, Oral Sarcopenia, and Hospital-Associated Oral Problems | doi:10.1007/s12603-020-1439-8 LK - |
| Ineligible design | Shirobe | 2025 | Predictive Factors for Swallowing Function Decline in Older Adults With Alzheimer's Disease: A 1-Year Longitudinal Study | doi:10.1111/joor.13912 LK - |
| not clear age | Singh | 2015 | Gender differences in the association between tooth loss and obesity among older adults in Brazil | doi:10.1590/S0034-8910.2015049005590 LK - |
| protocol | Qian | 2022 | Effects of Dental Implants and Nutrition on Elderly Edentulous Subjects: Protocol for a Factorial Randomized Clinical Trial | doi:10.3389/fnut.2022.930023 |
| wrong design | Söderström | 2022 | Oral nutritional supplement use is weakly associated with increased subjective health-related quality of life in malnourished older adults: a multicentre randomised controlled trial | doi:10.1017/S0007114521000805 |
| review | Soini | 2003 | Oral and nutritional status in frail elderly | doi:10.1111/j.1754-4505.2003.tb00314.x LK - |
| protocol | Sonawane and Mds | 2020 | “Evaluating salivary pH, uric acid, & C-reactive protein levels in completely edentulous patients before and after wearing complete dentures incorporated with and without 7.5% chitosan nanoparticles” – An interventional study |  |
| ineligible age | Stoichkov | 2023 | Dietary Intake of Vitamin D and Dental Caries Incidence in People with Overweight and Obesity | doi:10.2478/amb-2023-0002 LK - |
| wrong design | Stratidaki | 2024 | An Investigation of the Risk Factors Related to Frailty in Older Adults Receiving Home Care Services | doi:10.3390/nu16233982 LK - |
| review | Suominen | 2014 | Nutritional guidelines for older people in Finland | doi:10.1007/s12603-014-0509-1 LK - |
| Ineligible design | Takehara | 2021 | Appetite, oral health and weight loss in community-dwelling older men: an observational study from the Concord Health and Ageing in Men Project (CHAMP) | doi:10.1186/s12877-021-02169-y LK - |
| ineligible age | Takehara | 2024 | Weight Loss and Number of Present Teeth in Community-Dwelling Japanese Older Adults: A Cross-Sectional Study in Niigata | doi:10.1111/joor.13886 LK - |
| abstract only | Tan | 2019 | Nutrition, dietary modifications and palliative dental care in dementia patients: A case study | doi:10.1111/jgs.15898 LK - |
| ineligible age | TF | 2014 | Investigating tooth loss and associated factors among older Taiwanese adults | doi:10.1016/j.archger.2014.01.002 |
| ineligible age | TI Irawarti Rosli | 2019 | Association between oral health-related quality of life and nutritional status among older adults in district of Kuala Pilah, Malaysia | doi:10.1186/s12889-019-6867-1 |
| review | TN Jayasinghe | 2022 | Protein Intake and Oral Health in Older Adults-A Narrative Review | doi:10.3390/nu14214478 |
| ineligible age | Trachootham | 2019 | Tooth loss, protein intake and muscle mass in Thai elderly: The possible link |  |
| Ineligible design | Tuuliainen | 2020 | Oral Health and Hygiene and Association of Functional Ability: A Cross-Sectional Study Among Old Home Care Clients | doi:10.3290/j.ohpd.a43353 LK - |
| duplication | Tuuliainen | 2017 | Association of functioning and nutrition with oral health and hygiene among old home care clients |  |
| ineligible age | Uusitupa | 1992 | Chromium supplementation in impaired glucose tolerance of elderly: effects on blood glucose, plasma insulin, C-peptide and lipid levels | doi:10.1079/bjn19920078 |
| duplication | Dormenval | 1999 | Associations between self-assessed masticatory ability, nutritional status, prosthetic status and salivary flow rate in hospitalized elders | doi:10.1111/j.1601-0825.1999.tb00061.x |
| review | van der Pols-Vijlbrief | 2014 | Determinants of protein-energy malnutrition in community-dwelling older adults: A systematic review of observational studies | doi:10.1016/j.arr.2014.09.001 LK - |
| cannot retrieve | Velho | 2015 | Relationship between oral health and nutritional status in older patients admitted in a medical ward of a general hospital |  |
| ineligible age | Visvanathan and Ahmad | 2006 | Good oral health, adequate nutrient consumption and family support are associated with a reduced risk of being underweight amongst older Malaysian residents of publicly funded shelter homes Good oral health, adequate nutrient consumption and family support are associated with a reduced risk of being underweight amongst older Malaysian residents of publicly funded shelter homes |  |
| review | Valerie J. Horn | 1994 | Dental condition and weight loss in institutionalized demented patients | doi:10.1111/j.1754-4505.1994.tb01114.x |
| Ineligible design | Volpato | 2018 | Effects of oral amino acid supplementation on Multidimensional Prognostic Index in hospitalized older patients: a multicenter randomized, double-blind, placebo-controlled pilot study | doi:10.2147/CIA.S154998 |
| review | Walls | 2000 | Oral health and nutrition in older people | doi.org/10.1111/j.1752-7325.2000.tb03339.x |
| ineligible age | Wang | 2025 | Explaining the Association Between Number of Teeth and Frailty in Older Chinese Adults: The Chain Mediating Effect of Nutritional Status and Cognitive Function | doi:10.1111/ijn.70001 LK - |
| duplication | Watson | 2017 | The impact of dental status on perceived ability to eat certain foods, nutrient intake and nutritional status in older adults: UK National Diet and Nutrition Survey 2008-2012 | doi:10.1017/S0029665117001835 LK - |
| ineligible age | Watson | 2019 | The impact of dental status on perceived ability to eat certain foods and nutrient intakes in older adults: Cross-sectional analysis of the UK National Diet and Nutrition Survey 2008-2014 | doi:10.1186/s12966-019-0803-8 LK - |
| review | Weintraub | 2023 | Geroscience: Aging and Oral Health Research | doi:10.1177/08959374231200840 LK - |
| ineligible age | Wöstmann | 2008 | Influence of denture improvement on the nutritional status and quality of life of geriatric patients | doi:10.1016/j.jdent.2008.05.017 LK - |
| not clear age | Wu | 2025 | Association of oral health status with overweight and obesity in older adults aged_≥_65 years- evidence from Chinese communities | doi:10.1186/s12889-025-21546-5 LK - |
| ineligible age | Liu | 2024 | Association between life's essential 8 and periodontitis in U.S. adults | doi:10.1038/s41598-024-82195-z |
| Ineligible design | Xie | 2023 | Nutrition education with or without oral nutrition supplements has contrasting effects on nutrition status in older adults: a randomized controlled study | doi:10.1002/ncp.10898 |
| review | Algra | 2021 | The Association between Malnutrition and Oral Health in Older People: A Systematic Review | doi:10.3390/nu13103584 |
| Ineligible design | Gu | 2019 | Association between the number of teeth and frailty among Chinese older adults: a nationwide cross-sectional study | doi:10.1136/bmjopen-2019-029929 |
| ineligible age | Wang | 2025 | A Population-Based Study of the Association Among Dry Mouth, Vitamin B2, and Mortality | doi:10.1111/joor.13892 |
| Ineligible design | Yoshimura | 2024 | Triad of rehabilitation, nutrition support, and oral management improves activities of daily living and muscle health in hospitalized patients after stroke | doi:10.1016/j.clnesp.2024.08.018 |
| review | Yamanaka | 2010 | Comparison of the health condition between the 8020 achievers and the 8020 non-achievers | doi:10.1922/IDJ_1924Yamanaka05 LK - |
| ineligible age | Yang | 2024 | Associations between different triglyceride glucose index-related obesity indices and periodontitis: results from NHANES 2009–2014 | doi:10.1186/s12944-024-02192-z LK - |
| ineligible age | Ygnatios | 2023 | Food consumption is associated with frailty in edentulous older adults: evidence from the ELSI-Brazil study | doi:10.1590/1413-81232023287.12032022 LK - |
| cannot retrieve | Jung and Shin | 2008 | Oral Health, Nutrition, and Oral Health-Related Quality of Life: Among Korean Older Adults | doi:10.3928/00989134-20081001-09 |
| duplication | Yodogawa | 2022 | Erratum: Correction: Effects of food neophobia and oral health on the nutritional status of community-dwelling older adults (BMC geriatrics (2022) 22 1 (334)) | doi:10.1186/s12877-022-03013-7 LK - |
| ineligible age | Yoshihara | 2022 | Interaction between serum vitamin C levels and smoking on the periodontal condition in older adults | doi:10.1111/jre.12988 LK - |
| ineligible age | Yu | 2024 | The association between oral frailty and HbA1c among older adults with T2DM: the chain mediating effect of nutritional status and physical frailty | doi:10.1007/s41999-024-01081-z LK - |
| ineligible age | Yu | 2024 | The relationship between dysphagia and frailty among Chinese hospitalized older patients: a serial mediation model through self-perceived oral health and self-reported nutritional status | doi:10.1186/s12877-024-04684-0 LK - |
| review | Zelig | 2022 | Tooth Loss and Nutritional Status in Older Adults: A Systematic Review and Meta-analysis | doi:10.1177/2380084420981016 LK - |
| ineligible age | Zelig | 2019 | The Eating Experience: Adaptive and Maladaptive Strategies of Older Adults with Tooth Loss | doi:10.1177/2380084419827532 LK - |
| ineligible age | Zelig | 2024 | Dentition and weight status in community-dwelling older adults | doi:10.1111/ger.12747 LK - |
| ineligible age | Zelig | 2024 | A user-centered approach to the development of a diet education tool for older adults with tooth loss | doi:10.1111/ger.12697 LK - |
| review | Zelig | 2016 | Associations Between Tooth Loss, With or Without Dental Prostheses, and Malnutrition Risk in Older Adults |  |
| Ineligible design | Zhang | 2021 | Effect of an individualised nutritional intervention on gestational diabetes mellitus prevention in a high-risk population screened by a prediction model: study protocol for a multicentre randomised controlled trial | doi:10.1186/s12884-021-04039-2 |
| ineligible age | Zhang | 2022 | The Association between Number of Teeth and Cognitive Frailty in Older Adults: A Cross-Sectional Study | doi:10.1007/s12603-022-1783-y LK - |
| ineligible age | Zhao | 2023 | Tooth loss, body mass index and cognitive function among middle-aged and older adults in China: Does gender matter? |  |
| ineligible age | Zheng | 2016 | Nutrition Status, Nutrition Support Therapy, and Food Intake are Related to Prolonged Hospital Stays in China: Results from the NutritionDay 2015 Survey |  |
| ineligible age | Zhu | 2022 | Association of high-density lipoprotein cholesterol and periodontitis severity in Chinese elderly: a cross-sectional study | doi:10.1007/s00784-022-04439-4 LK - |
| review | Maria Antoniadou and Theodoros Varzakas | 2021 | Breaking the vicious circle of diet Breaking the vicious circle of diet, malnutrition and oral health for the independent elderly |  |
